# A comprehensive AMR genotype–phenotype database (CABBAGE)

**DOI:** 10.1093/nar/gkag780

**Published:** 2026-08-24

**Authors:** Emily Dickens, Romain Derelle, Robert Beardmore, Anita Suresh, Swapna Uplekar, Andrey G Azov, Tatiana A Gurbich, Bilal El Houdaigui, Jon Keatley, Sofiia Ochkalova, Orges Koci, Nadim M Rahman, Anu Shivalikanjli, Andrea Winterbottom, Galabina Yordanova, Helen Parkinson, Andrew D Yates, Robert D Finn, John A Lees, Leonid Chindelevitch

**Affiliations:** MRC Centre for Global Infectious Disease Analysis, School of Public Health, Imperial College London, London, W12 0BZ, United Kingdom; MRC Centre for Global Infectious Disease Analysis, School of Public Health, Imperial College London, London, W12 0BZ, United Kingdom; Biosciences, University of Exeter, Exeter, EX4 4QD, United Kingdom; University Hospital Heidelberg, Heidelberg, 69120, Germany; Foundation for Innovative New Diagnostics (FIND), Geneva, 1218, Switzerland; European Molecular Biology Laboratory, European Bioinformatics Institute, Wellcome Genome Campus, Hinxton, CB10 1SD, United Kingdom; European Molecular Biology Laboratory, European Bioinformatics Institute, Wellcome Genome Campus, Hinxton, CB10 1SD, United Kingdom; European Molecular Biology Laboratory, European Bioinformatics Institute, Wellcome Genome Campus, Hinxton, CB10 1SD, United Kingdom; European Molecular Biology Laboratory, European Bioinformatics Institute, Wellcome Genome Campus, Hinxton, CB10 1SD, United Kingdom; European Molecular Biology Laboratory, European Bioinformatics Institute, Wellcome Genome Campus, Hinxton, CB10 1SD, United Kingdom; European Molecular Biology Laboratory, European Bioinformatics Institute, Wellcome Genome Campus, Hinxton, CB10 1SD, United Kingdom; European Molecular Biology Laboratory, European Bioinformatics Institute, Wellcome Genome Campus, Hinxton, CB10 1SD, United Kingdom; European Molecular Biology Laboratory, European Bioinformatics Institute, Wellcome Genome Campus, Hinxton, CB10 1SD, United Kingdom; European Molecular Biology Laboratory, European Bioinformatics Institute, Wellcome Genome Campus, Hinxton, CB10 1SD, United Kingdom; European Molecular Biology Laboratory, European Bioinformatics Institute, Wellcome Genome Campus, Hinxton, CB10 1SD, United Kingdom; European Molecular Biology Laboratory, European Bioinformatics Institute, Wellcome Genome Campus, Hinxton, CB10 1SD, United Kingdom; European Molecular Biology Laboratory, European Bioinformatics Institute, Wellcome Genome Campus, Hinxton, CB10 1SD, United Kingdom; European Molecular Biology Laboratory, European Bioinformatics Institute, Wellcome Genome Campus, Hinxton, CB10 1SD, United Kingdom; European Molecular Biology Laboratory, European Bioinformatics Institute, Wellcome Genome Campus, Hinxton, CB10 1SD, United Kingdom; MRC Centre for Global Infectious Disease Analysis, School of Public Health, Imperial College London, London, W12 0BZ, United Kingdom

## Abstract

Addressing the growing threat of antimicrobial resistance (AMR) requires the development of large-scale resources that link bacterial genomic data with phenotypic AMR profiles. Such datasets are essential for advancing genotype-based predictions of resistance to uncover novel resistance mechanisms, as well as identifying and tracking global trends. Here, we describe the development of the “Comprehensive Assessment of Bacterial-Based AMR prediction from GEnotypes” (CABBAGE) database, linking bacterial genomes to associated antibiotic susceptibility data and relevant metadata across WHO Bacterial Priority Pathogens, sourced from both publications and existing databases, and curated into a format that is compatible with, and extends, both NCBI and ENA formats. The resulting CABBAGE database, comprising over 170 000 unique sequenced isolates and approximately 1.7 million genome–phenotype pairs linked to extensive metadata, represents the largest database of its kind, consolidating existing AMR phenotype–genotype data into a single unified format. CABBAGE encompasses a broad range of antimicrobials, facilitating the analysis of global resistance trends as well as benchmarks of genotype-to-phenotype predictive methods, and empowering further research uses. The database is freely accessible via the Antimicrobial Resistance Portal at EMBL-EBI and is currently being integrated with the BioSample database, enabling easy access for the AMR research community.

## Graphical Abstract

**Graphical Abstract:**
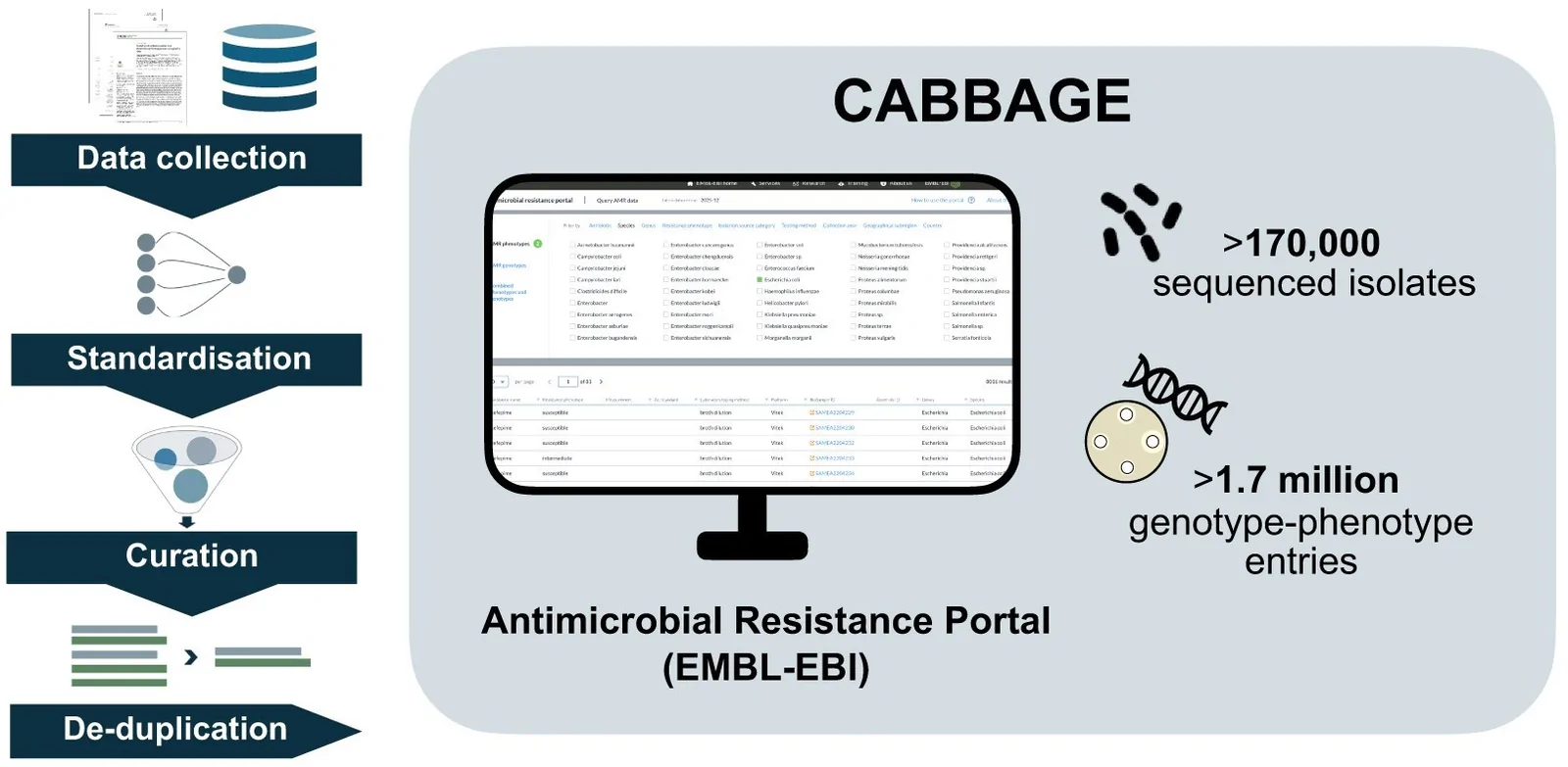

## Introduction

Antimicrobial resistance (AMR) poses a substantial risk to global health, resulting in increased morbidity, mortality, and healthcare associated costs. Addressing this challenge requires a multifaceted approach, ranging from clinical interventions such as enhanced antibiotic stewardship and diagnostics, to pharmaceutical initiatives, including the development of novel antimicrobials. Among these, advances in whole-genome sequencing (WGS) have raised the possibility of using genomics-based diagnostics alongside, or as a replacement for, traditional antibiotic susceptibility tests (ASTs). However, while numerous AMR genes have been identified, discordance between genotypic predictions and phenotypic resistance is observed across numerous studies [1–3]. A key step in addressing this discordance is the development of large-scale, curated datasets integrating AMR phenotypes with corresponding genomic data to support machine learning prediction and the identification of novel resistance mechanisms.

The World Health Organization (WHO) has identified a list of bacterial pathogens which are a priority for new antibiotic development, encompassing 26 bacterial pathogen–antibiotic combinations in 2017 [4], revised to 24 in 2024 [5]. However, while the development of novel antimicrobials remains critical in addressing the growing threat of AMR, this strategy is often viewed as unsustainable due to the substantial costs associated with drug development and the risk of resistance developing soon after new antimicrobials are introduced into clinical use. An alternative approach is to maximize the effectiveness of existing antimicrobials with appropriate diagnostics and antimicrobial stewardship efforts [6, 7]. This could involve making treatment decisions informed by aggregated phenotypic and genotypic AMR data; however, the availability of clinically derived AMR data varies between countries and clinical settings [8], particularly in low- and middle-income countries that have limited representation in AMR surveillance programs [9]. In addition, while WGS is increasingly used for AMR surveillance, there are multiple barriers hindering widespread adoption, such as cost, infrastructure, and bioinformatics requirements [10–12]. Nevertheless, a wealth of genotypic and phenotypic information relevant to AMR already exists in various formats within public repositories.

A number of AMR databases have been developed to support research and surveillance efforts. Key resources include the Bacterial and Viral Bioinformatics Resource Center (BV-BRC) [13], the NCBI BioSample Antibiograms database (https://www.ncbi.nlm.nih.gov/biosample/docs/antibiogram/) and the National Database of Antibiotic Resistant Organisms (NDARO) (https://www.ncbi.nlm.nih.gov/pathogens/antimicrobial-resistance/), all of which provide extensive bacterial genomic data linked to antimicrobial susceptibility data. Similarly, PubMLST [14], although primarily focusing on the provision of MLST information, also integrates bacterial population genomic data with phenotypic information, while platforms such as Microreact [15] and PathogenWatch (https://pathogen.watch) enable the visualization of global AMR data alongside accompanying genotypic data when available. National level surveillance is also supported by agencies in the United States such as the Centres for Disease Control and Prevention (CDC) [16], which reports AMR trends, and the National Antimicrobial Resistance Monitoring System (NARMS) [17], which focuses on foodborne bacteria. Additionally, the European COMPARE Consortium [18] has created an AMR data hub consisting of over 2500 sequenced isolates linked to structured phenotypic data. However, despite the availability of these resources, current AMR data remain largely fragmented across various institutions and regions, limiting its collective influence. There is, therefore, a need to develop a single, harmonized AMR database that integrates existing data, standardizes data formats, and incorporates expert curation. Publications often also contain genotype–phenotype data and metadata that has not been included in any of the aforementioned resources, and can therefore provide an additional valuable source of information on AMR phenotypes and matching genotypes. A key challenge is that they can rarely be processed in a fully automated way.

The work presented in this study aimed to combine all available isolates of species belonging to the WHO Priority Pathogens with both genomic and antibiotic susceptibility testing data, drawing on two sources: an analysis of existing databases containing such datasets, as well as additional large AMR genotype–phenotype datasets extracted from the literature, followed by a process of data standardization and curation. We highlight cross-database redundancy across the aforementioned databases that, although anticipated, has only now been fully characterized following their integration, revealing the extensive conflicts between data records and de-duplication challenges. The end result is the “Comprehensive Assessment of Bacterial-Based AMR prediction from GEnotypes” (CABBAGE) database, comprising over 170 000 unique sequenced isolates and approximately 1.7 million genome–phenotype pairs linked to extensive metadata. Here, we present an overview of CABBAGE, including the availability of data among different antibiotics and pathogens, the spatio-temporal data distribution, as well as a comparative analysis with the Pfizer Antimicrobial Testing Leadership and Surveillance (ATLAS) database (www.atlas-surveillance.com), a global AMR dataset encompassing over 900 000 isolates with associated antimicrobial susceptibility data, but no genotypic data aside from the presence/absence of a limited number of *β*-lactamase genes. Lastly, we describe how to access the CABBAGE dataset via the Antimicrobial Resistance Portal at EMBL-EBI (https://www.ebi.ac.uk/amr).

CABBAGE is a valuable resource, with applications ranging from advancing genotype-to-phenotype machine learning models that further our understanding of AMR mechanisms, to improvements in clinical diagnostics and global AMR surveillance. Additionally, the Antimicrobial Resistance Portal encourages data sharing by data producers in a standardized format, facilitating the long-term growth of CABBAGE.

## Materials and methods

### Data collection

A literature review was carried out with the aim of identifying and extracting data from publications containing at least 100 sequenced isolates on the WHO Bacterial Priority Pathogens List, with AMR phenotypes available for at least one drug. The search terms used are summarized in Supplementary Table S1. Note that this 100 isolate threshold was adjusted for some pathogens to account for their over- or under-representation in the literature, as detailed in the Supplementary Methods (S1.1). For each dataset with publicly available data, tables containing phenotypes as well as the accession numbers for the genotypes were extracted and standardized into a CSV format. These files were then processed via custom scripts, available at https://github.com/Leonardini/CABBAGE, to identify all relevant information on the genotype, phenotype, and metadata. A flow chart illustrating the literature review process is provided in Supplementary Fig. S1.

In addition to the datasets extracted from the literature, we identified nine public databases (Microreact, NARMS, NDARO, Pathogenwatch, BV-BRC [formerly known as PATRIC], PubMLST, COMPARE, CDC, and NCBI antibiograms) as containing relevant information and therefore extracted their data. The number of entries per pathogen contributed by each database is listed in Supplementary Table S2 and summarized in Supplementary Table S3.

As the genomic component of CABBAGE entries is structured around BioSample IDs, all sequencing run IDs, sample IDs, and genome accession IDs were converted to BioSample IDs. Missing values in these genomic fields were then retrieved using BioSample IDs as the central reference. In addition, to improve the completeness of metadata fields, isolate metadata were retrieved for entries with missing values, again using BioSample IDs as the reference.

### Data standardization

To ensure comparability across datasets obtained from different sources, the data were mapped to a set of 27 standardized fields (Fig. 2), with each entry being defined by a pair of BioSample ID (genotype) and antibiotic name (phenotype). A key step in the data standardization process involved the harmonization of antibiotic names and their extraction from abbreviations as, for instance, a single abbreviation may refer to numerous different antibiotics across different datasets. To address this, antibiotic names were derived from the antibiotic abbreviations provided in the source dataset, referring to the paper’s full text when necessary, and standardized using the name list employed by NCBI antibiograms.

Further data standardization steps include, but are not limited to, the conversion of numerical phenotypes (e.g. 0, 1) to their corresponding classification (e.g. susceptible and resistant), the standardization of country names based on ISO 3166-1 alpha-3 codes, standardizing both broth microdilution and macrodilution to broth dilution, and standardizing the host to either “*Homo sapiens*” or “other (non-clinical isolate)”. A full description of the data standardization process is included in the Supplementary Methods (S1.5).

### Data curation

A series of curation steps were performed to remove or correct inconsistent entries (Supplementary Fig. S2). Only those entries that were inconsistent or conflicting were manually curated. Pathogens not recorded in the WHO Bacterial Priority Pathogens List were excluded, and several species were renamed for consistency (e.g. *Salmonella* Typhimurium was changed to *Salmonella enterica*). Particular attention was paid to the curation of genomic identifiers (the genomic component of CABBAGE), as well as measurement values and typing methods (the phenotypic component of CABBAGE).

#### Genomic data curation

During data collection and the subsequent conversion of genomic identifiers to BioSample IDs, we identified cases where multiple genomic identifiers within a single entry did not point to the same BioSample ID (e.g. a sequencing run ID and a genome accession ID corresponding to different BioSample IDs). As the correct BioSample ID could not be determined, these entries were excluded. We also identified multiple BioSample IDs pointing to the same sequencing run ID. The corresponding entries were also excluded as these cases likely correspond to pooled isolate sequencing.

#### Phenotypic data curation

Minimum inhibitory concentration (MIC) values were examined for potential errors that were subsequently removed, for example, large values inconsistent with standard doubling dilution ranges (e.g. 1 000 000). Typing methods associated with anomalous diffusion zone diameter values (e.g. small decimal values) or other irregular MIC values (e.g. series of integers outside standard dilution ranges) were manually checked against the original data sources. When errors were confirmed, both the method and the measurement unit were corrected. Original data sources were also checked to retrieve corresponding typing methods in cases where a measurement value was present without a unit and method. The typing methods associated with the datasets obtained from BV-BRC are shown in Supplementary Table S5. Further curation steps addressed anomalies in metadata fields, such as high values of host age (e.g. 999) and improbable collection years (e.g. 1800) that were removed.

The final curation stage involved the de-duplication of entries, including entries that were either identical or shared the same pair BioSample ID/antibiotic name but differed in measurement value or phenotype. In the case of identical entries, duplicates were removed, and conflicting entries that could not be resolved by checking the original sources were also excluded. A full description of the data curation process is included in the Supplementary Methods (S1.6).

### Final processing

In cases where both a measurement value and categorical phenotype (e.g. resistant [R], intermediate [I], or susceptible [S]) are recorded, the phenotype is often derived using outdated or custom breakpoints. Additionally, many entries include a measurement value, but lack a corresponding phenotype. To address this, we incorporated updated phenotypes in the “Updated phenotype CLSI” and “Updated phenotype EUCAST” fields by applying the current 2025 breakpoint criteria from both CLSI and EUCAST, inferred using the AMR (for R) package v3.0.0 [19]. These phenotypes will be updated using the most recent breakpoint criteria in future versions of CABBAGE.

The CABBAGE database then underwent a series of consistency checks to ensure that values within each entry were consistent across fields. For instance, a testing standard can only be reported in the presence of a phenotype, a platform value should have a corresponding typing method, a measurement unit is required if a measurement value is provided alongside a typing method, and each entry must contain both a BioSample ID and either a measurement value or a categorical resistance phenotype.

### Genome assembly and annotation

Genome annotation was conducted on genomes previously submitted to the European Nucleotide Archive and on newly assembled genomes from the AllTheBacteria collaboration [20]. Genomes were assessed using CheckM2 with 1269 genomes filtered due to a completeness score <50 or contamination score >5 [21]. After these quality control steps, if multiple genome assemblies were linked to the same BioSample ID, genome annotation was performed using the most recent GCA-accessioned genome assembly. If, however, no GCA-accessioned genome assemblies were available, the most recent ERZ-accessioned genome assembly was used. Each genome in the CABBAGE data set was annotated using the pipeline mettannotator [22], which includes AMR annotation from AMRFinderPlus [23], providing a unified and consistent annotation set across all CABBAGE genomes.

### Database accessibility

CABBAGE is available via the Antimicrobial Resistance Portal at EMBL-EBI (https://www.ebi.ac.uk/amr). The resource will be updated on a regular basis to incorporate new datasets and functionality. For example, a key priority is the development of an automated pipeline to facilitate the integration of data from BioSamples and ENA, thereby supporting the ongoing expansion of the database as such data becomes available. Additionally, all data will be retained and archived to support long-term accessibility. All data, including genome annotations, are made available via the EMBL-EBI FTP site (https://ftp.ebi.ac.uk/pub/databases/amr_portal) under a CC-BY 4.0 license. The data are also available in apache parquet format for raw download. Antibiograms have been deposited alongside each BioSample record. Assembled genomes are available via the European Nucleotide Archive.

## Results

### Antimicrobial resistance portal

**Figure 1.**
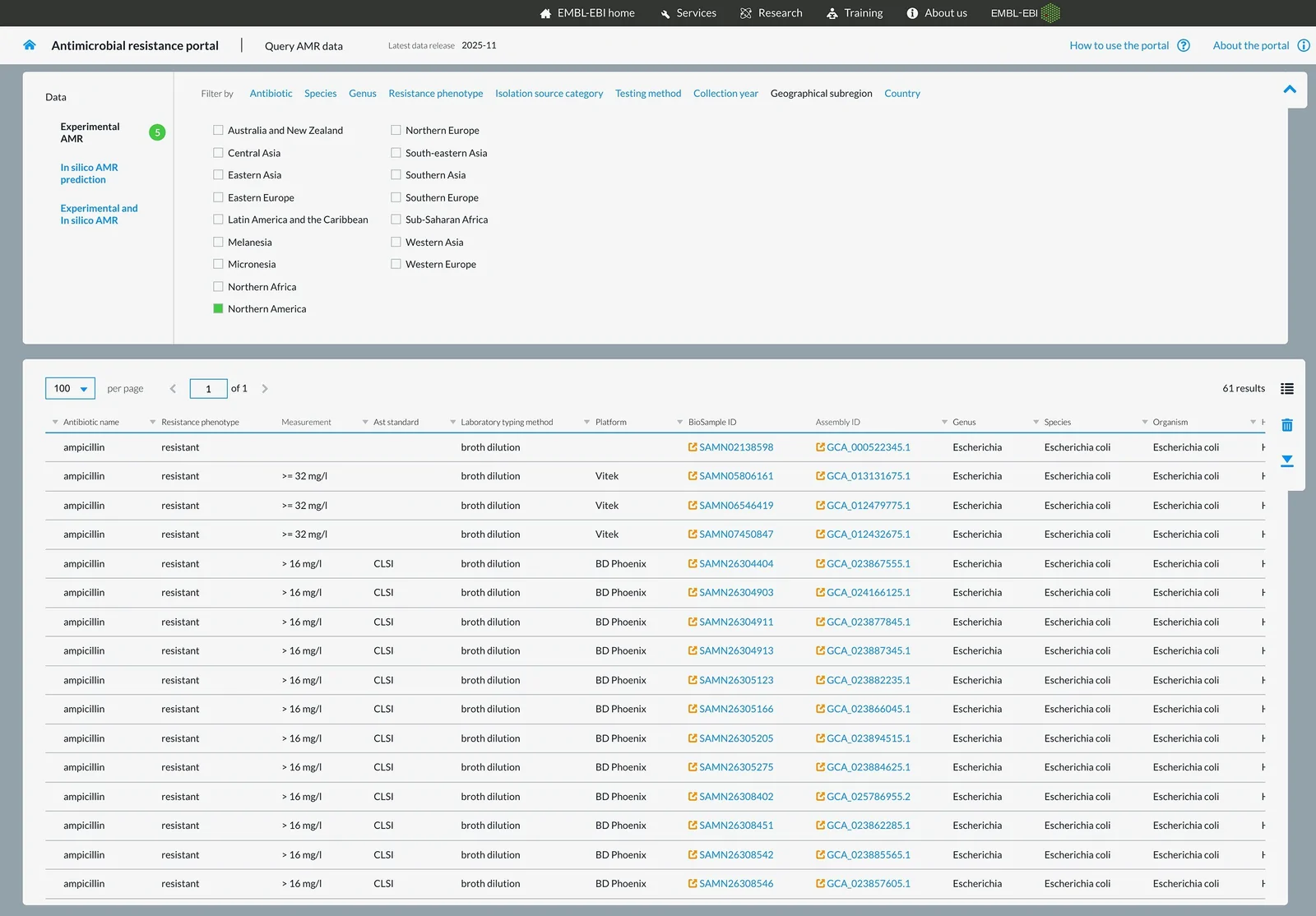
Screenshot of the Antimicrobial resistance portal, showing the CABBAGE database filtered by antibiotic, species, isolation source category, and geographical region.

The Antimicrobial resistance portal provides a user-friendly interface to explore CABBAGE and consists of three main data resources: “AMR phenotypes”, consisting of antibiogram records; “AMR genotypes”, consisting of *in silico* annotations from AMRFinderPlus; and “Combined phenotypes and genotypes”, a merged dataset of phenotypes and genotypes. Within each resource, data can be filtered based on key data attributes, including species, genus, and resistance phenotype, among others (Fig. 1). Once filtered, data can be downloaded in CSV format, or each individual data set can be downloaded for offline processing.

### Overview of the CABBAGE database

**Figure 2.**
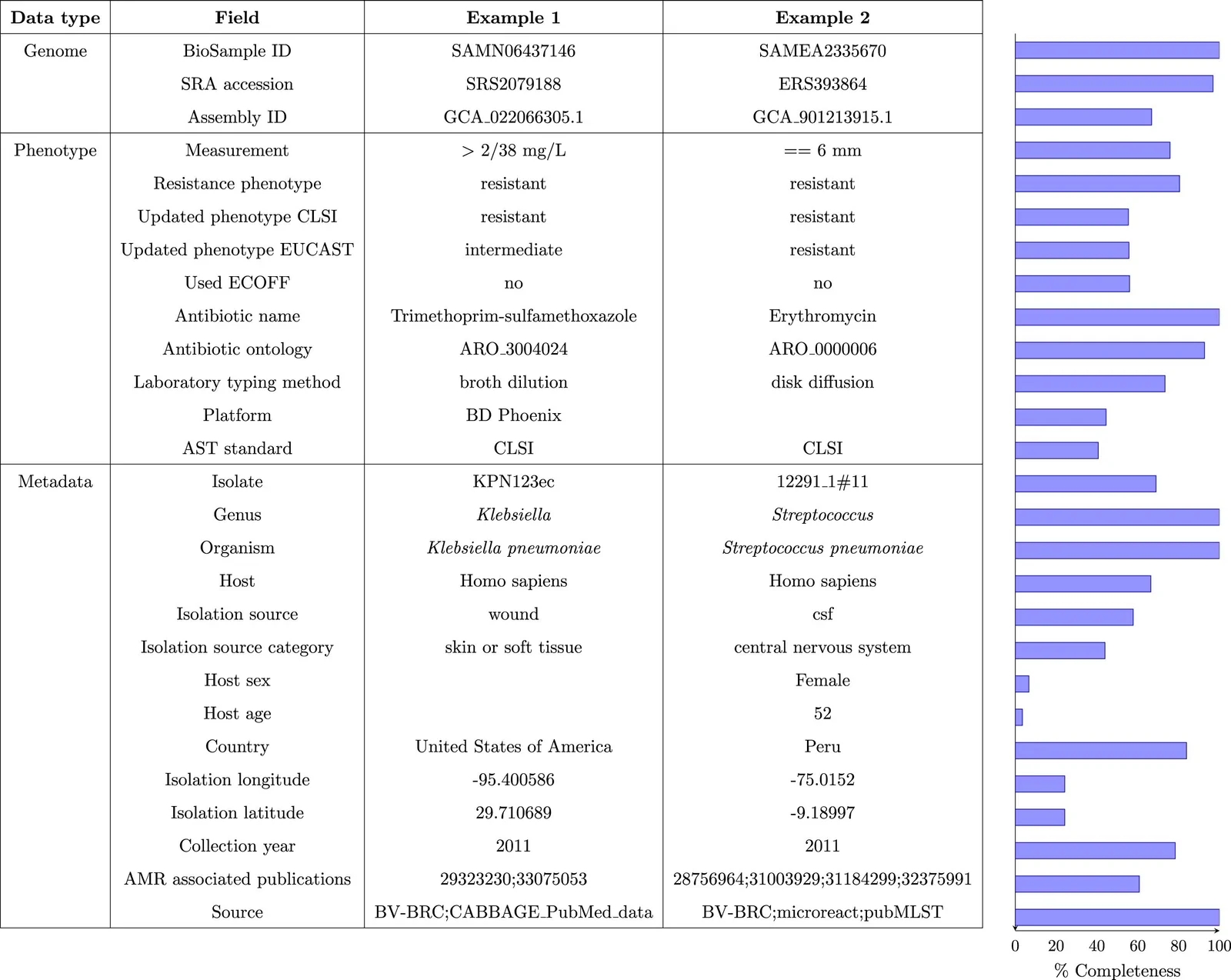
CABBAGE database fields, grouped into genomic, phenotypic, and metadata categories. Two examples of CABBAGE entries are provided, as well as the percentage of completeness of each field on the right side.

In total, CABBAGE contains antibiotic susceptibility data and associated metadata for 170 750 sequenced isolates, with an average of 10 *±* 6 (SD) AST results per isolate. The database contains *> *1.7 million entries, each entry being defined by a BioSample-antibiotic combination with an associated AST result. The AST result can be represented by a measurement value and/or a categorical phenotype (e.g. S, I, or R). Both a measurement value and a categorical phenotype are recorded for *> *964 000 entries, and both a measurement value and an updated phenotype, which we derived by comparing the measurement value to the latest CLSI and EUCAST breakpoints, are recorded for *> *1.1 million entries (Supplementary Table S4). Figure 2 summarizes field completeness alongside two examples of CABBAGE entries. While the fields defining entries are 100% complete, some metadata fields, such as host sex and age, are sparsely populated but are retained in the anticipation that coverage may improve as the database expands.

**Figure 3.**
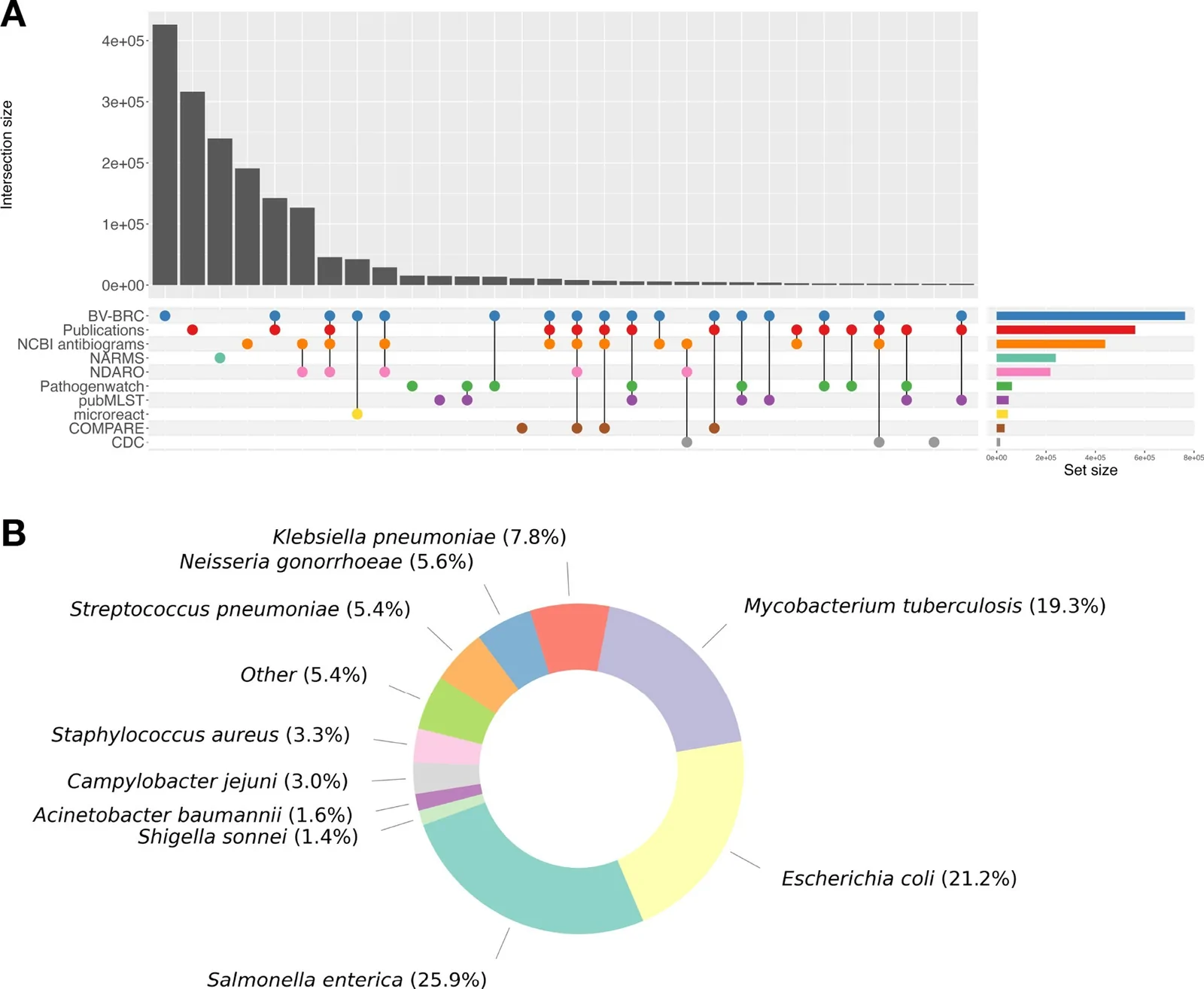
(**A**) Sources of CABBAGE data, in numbers of entries. Only sets with a minimum intersection size of 2000 are displayed. The full UpSet plot is provided in Supplementary Fig. S3. (**B**) Distribution of the 10 most common pathogens in CABBAGE. All remaining species are grouped under “Other”. Percentages represent the number of entries (pathogen–antibiotic pairs). The antibiotic testing frequency across the 10 most common pathogens in CABBAGE is displayed in Supplementary Fig. S5.

The number of entries obtained from, and shared between, different data sources is summarized in Fig. 3A. BV-BRC is the largest single data source, contributing just over 764 000 entries, followed by datasets derived from our literature review (89 publications), with over 560 000 entries. We observed high redundancy between data sources, which allowed us to identify and remove problematic entries during the database deduplication process. BV-BRC and the data sourced from publications have the largest number of entries in common with just each other (*> *142 000), followed by NCBI antibiograms and NDARO (*> *126 000). The number of entries associated with a single, multiple, or no PubMed ID is summarized in Supplementary Fig. S4.

Finally, the data are not evenly distributed across pathogens, with three species representing more than half of entries: *Salmonella enterica* (25.9%), *Escherichia coli* (21.2%), and *Mycobacterium tuberculosis* (19.3%) (Fig. 3B). In contrast, other pathogens are under-represented, with some species being represented by a single unique isolate: *Enterobacter cancerogenus, Serratia fonticola, Enterobacter mori, Enterobacter soli, Serratia nematodiphila, Providencia alcalifaciens, Proteus alimentorum*, and *Enterobacter sichuanensis*.

### Spatio-temporal distribution of CABBAGE data

**Figure 4.**
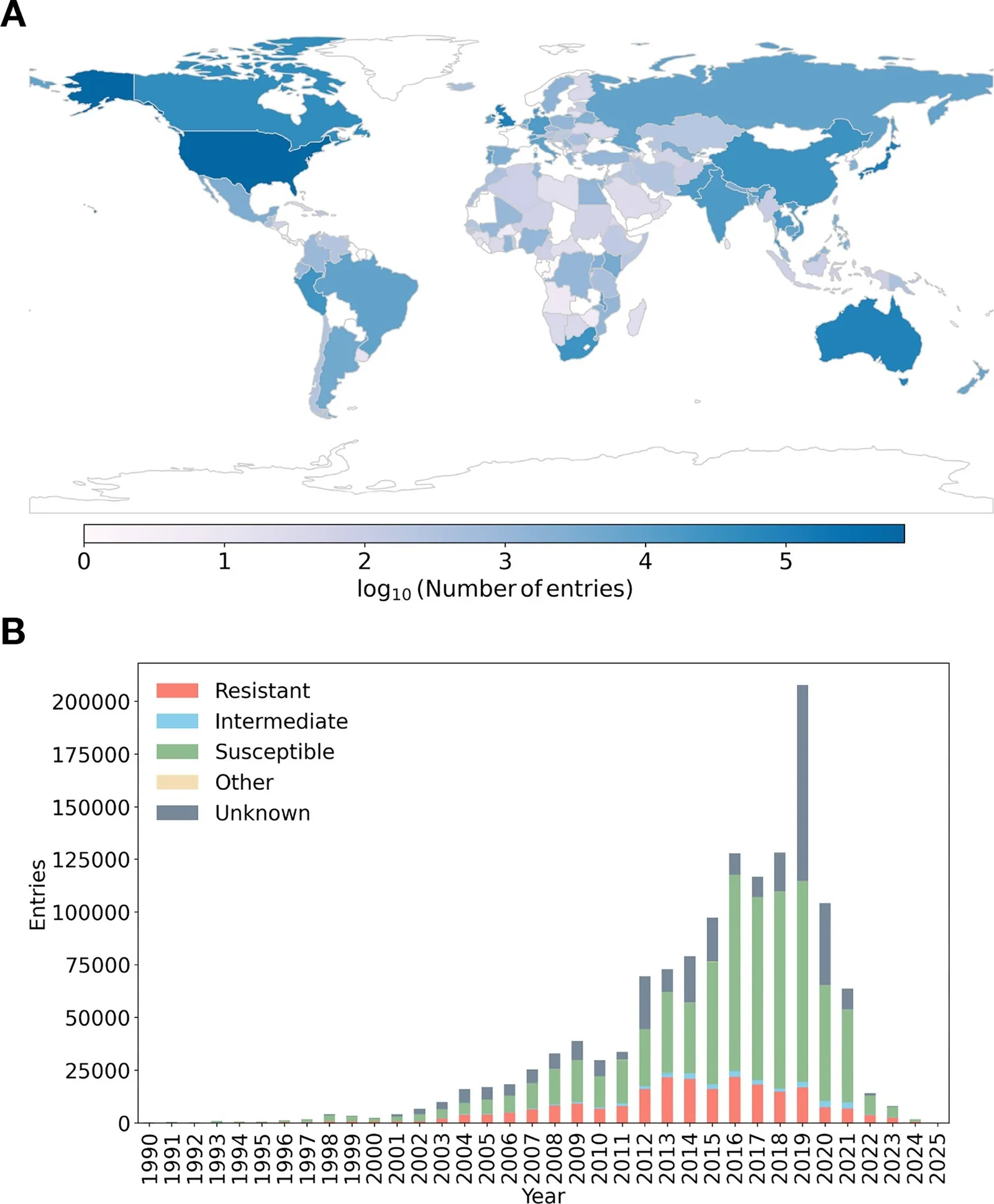
(**A**) Geographical distribution of entries (log_10_). Countries that are not represented in CABBAGE are shown in white. (**B**) Distribution of CABBAGE entries per collection years. The category “other” includes susceptible-dose dependent and non-susceptible. Frequencies of each categorical phenotype per year are shown in Supplementary Fig. S7B.

As expected, we observed substantial geographical disparities in the AMR data, with some regions, such as Central Africa and Central America, being sparsely sampled (Fig. 4A). The United States has the largest data coverage (*> *698 000 entries), in part due to their formidable AMR surveillance programs and associated databases (NARMS, NDARO, and CDC). Additionally, these inconsistencies in global data distribution may reflect differences in how readily organizations make data available following collection, as well as the under-representation of low- and middle-income countries in AMR surveillance programs due to resource limitations [24, 25]. Variations are also seen in the frequency of resistance phenotypes between different countries, as illustrated in Supplementary Fig. S6. However, caution should be taken when comparing resistance frequencies between countries due to varying levels of data coverage and ascertainment bias due to the fact that not all isolates are sequenced, and in some settings, sequencing is prioritized for those isolates that are phenotypically resistant to at least one antibiotic [26].

New AMR datasets are being published at an increasingly rapid pace, as illustrated in Fig. 4B. There is a marked increase in the number of entries from approximately the year 2000 onwards, likely reflecting advances in WGS, AMR surveillance, and data sharing. However, we also observed a sharp decline in the number of CABBAGE entries corresponding to recent years. This decline may be purely technical, reflecting a lag in the incorporation of recent data into public databases. Alternatively, it could indicate a genuine reduction in global AMR data collection, resulting from the substantial impact of the COVID-19 pandemic on AMR surveillance programs and academic research [27–29]. Future updates of the CABBAGE database incorporating more recent data sources will help disentangle the relative contributions of these two, not mutually exclusive, hypotheses.

### Antibiotic resistance frequency in CABBAGE

**Figure 5.**
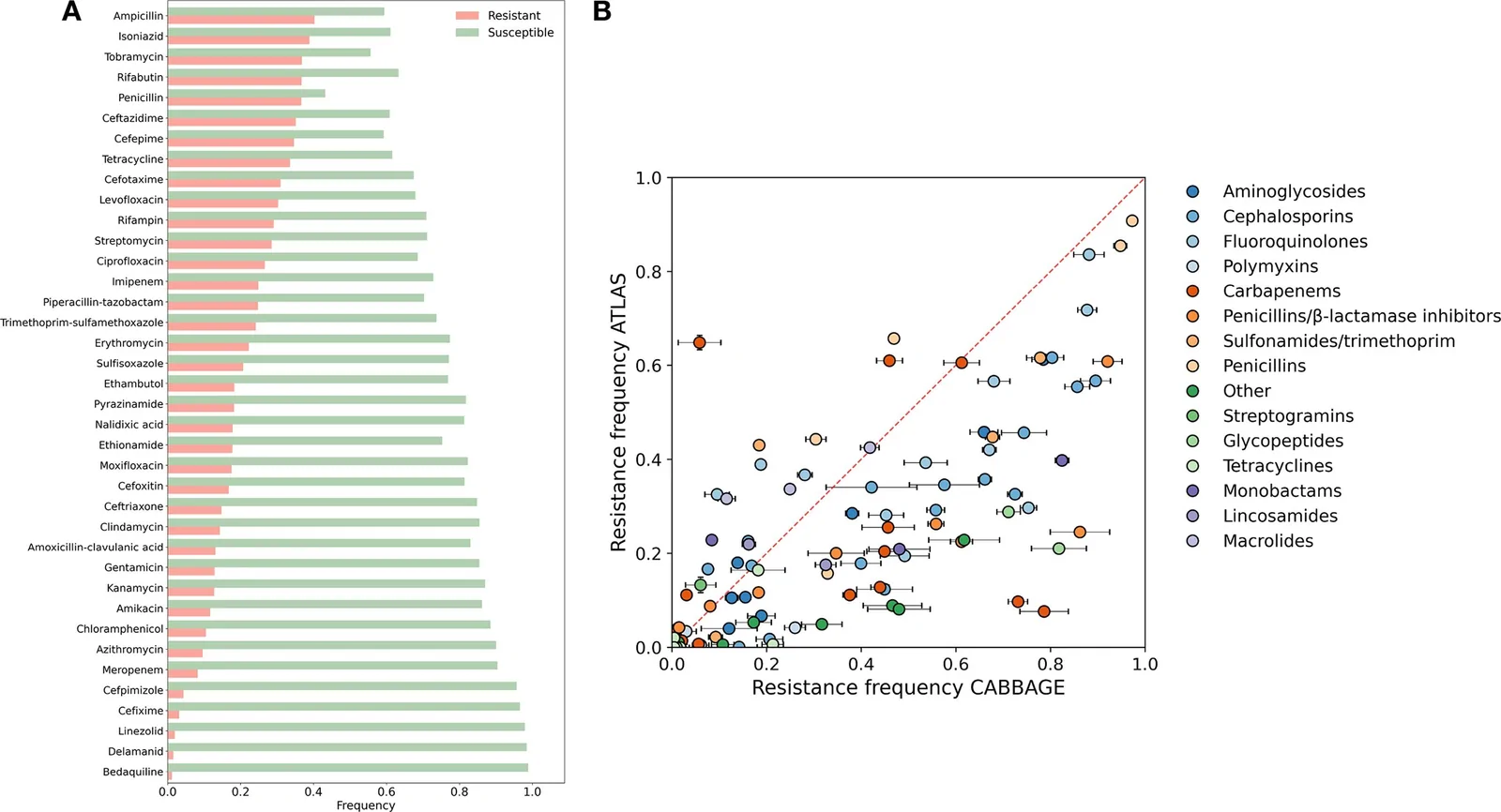
(**A**) Frequencies of resistant (red bars) versus susceptible (green bars) phenotypes across the CABBAGE dataset, by antibiotic. We used here the phenotypes extracted from the original data source, not the updated phenotypes, and the data were restricted to antibiotics with at least 10 000 entries in CABBAGE. Absolute numbers of susceptible and resistant entries are displayed in Supplementary Fig. S8. The number of isolates tested per antibiotic is shown in Supplementary Fig. S9. (**B**) Frequencies of resistance in CABBAGE versus ATLAS. Each data point represents a unique pathogen–antibiotic pair present in both datasets, restricted to data collected between 2004 and 2023 to reflect the range of collection dates in ATLAS. A scatter plot without this date restriction is presented in Supplementary Fig. S10. Pathogen–antibiotic pairs with at least 100 entries in both CABBAGE and ATLAS are shown. Error bars represent 95% CI and colours are representative of different antibiotic classes. The CABBAGE phenotypes presented here represent the phenotypes derived from the original datasets, rather than the updated phenotypes.

The percentage of resistant isolates varies widely between drugs (Fig. 5A). Among antibiotics with the greatest data coverage (at least 10 000 isolates), ampicillin showed the highest resistance frequency (40%), whereas bedaquiline had the lowest (1.2%). These differences likely reflect both the time since each drug’s introduction and their overall levels of clinical use. However, the CABBAGE dataset as a whole is expected to be biased toward resistant phenotypes (22% of CABBAGE entries with a categorical phenotype are resistant), since such isolates are more often sequenced and reported.

To evaluate this potential bias, we compared the resistance frequencies of pathogen–antibiotic pairs shared between CABBAGE and the ATLAS database (104 pairs; Fig. 5B). ATLAS serves as a useful comparator because it compiles results from large global surveillance studies (*> *900 000 isolates). Logistic regression analysis revealed that resistance was significantly more frequent in CABBAGE than in ATLAS (OR = 1.6, *p < 0.001*). Nonetheless, this trend was not universal: 66 pathogen–antibiotic pairs showed significantly higher resistance in CABBAGE, while 29 showed greater susceptibility (Fisher’s exact test, FDR adjusted *p < 0.05*).

## Discussion

Comprehensive, large-scale datasets integrating bacterial genome sequences with associated phenotypic antibiotic susceptibility data are essential for the development of genotype-to-phenotype predictive models to further our understanding of AMR mechanisms and to inform the design of improved diagnostics. Although a number of databases provide such information, up until now these data have not been collated and processed into a single uniform format, limiting its collective analysis. A substantial volume of data also remains in the public domain across multiple publications, with more being added on a weekly basis. To address this challenge, we developed CABBAGE, a comprehensive, global, curated genotype–phenotype AMR database for WHO Bacterial Priority Pathogens. CABBAGE is the largest database of its kind, freely available via the Antimicrobial Resistance Portal at EMBL-EBI (https://www.ebi.ac.uk/amr), facilitating user-friendly access.

Through an extensive literature review and examination of public databases, we extracted data on *> *170 000 unique sequenced isolates with associated phenotypic AMR data, totalling *> *1.7 million genome–phenotype pairs. In addition, each entry is associated with metadata where available, such as the isolate collection date, isolation source, and geographic location. The data were standardized into a uniform format and curated, where necessary, to improve consistency, accuracy, and completeness. CABBAGE constitutes a wealth of information that facilitates benchmarks of genotype-to-phenotype predictive models, with the potential to enhance our understanding of the genetic mechanisms of AMR, including the identification of novel resistance determinants. Additionally, CABBAGE enables the analysis of spatio-temporal resistance trends for AMR surveillance and can be used to enhance clinical diagnostic tools, such as genome-based antimicrobial susceptibility testing.

Since the preprocessing pipeline may influence the downstream inference [30, 31], we recommend that data producers make genotype data available in raw format, namely as sequence reads submitted to the ENA. Additionally, antibiogram data should ideally be submitted to BioSamples. As part of this work, we have created new guides to help data generators archive their data: https://www.ebi.ac.uk/amr/amr_submission_guide/. By submitting both genotype and phenotype data to biomedical data archives, this achieves longevity of storage in a uniform format, maximizing reusability, and avoiding being tied to a single research project. Prediction methods would likely need to first pre-process raw read data into genomic features, which is important to do in a consistent manner across the dataset. Assemblies, gene calls or variant calls agreed by the community are a useful resource for reusers of these data, and our consistent assembly and annotation of data was designed to save computational time and improve consistency for downstream users of CABBAGE.

The data within CABBAGE exhibits multiple layers of heterogeneity, for example, phenotypes vary from quantitative MICs and disk diffusion data obtained from a variety of AST methods, to S/I/R classifications obtained using guidelines that may be outdated. We addressed this by providing additional updated phenotypes based on current EUCAST and CLSI guidelines. The species in CABBAGE are also unevenly distributed, with *S. enterica* alone accounting for just over 25% of all entries, and at the other extreme, a number of species are only represented by a single isolate. Similar heterogeneities are found for the antibiotics, publications, countries represented, and the years of collection. We specifically highlight a lag in the availability of new datasets over recent years, and hypothesize that this could be due to the impact of the COVID-19 pandemic on AMR research, a delay in the incorporation of new data into databases, or a combination of both these factors.

Lastly, we note that the fraction of resistant isolates is likely to be over-estimated, as many groups tend to perform AST on all isolates of interest, but only sequence those that are resistant. We illustrate this bias towards resistance through a comparative analysis of CABBAGE against the AMR dataset ATLAS, showing that, overall, there is a greater likelihood of resistance in CABBAGE relative to ATLAS. It is possible that CABBAGE could be used to systematically identify gaps in surveillance, even though, depending on the sampling methodology and the resources available, such gaps may or may not be easy to fill. Future updates to CABBAGE will focus on extending the database, particularly for under-represented species, and applying the most recent WHO Bacterial Priority Pathogen List (2024).

In summary, CABBAGE consolidates data from existing resources into a single, unified database. Through the integration, standardization and curation of information that was previously fragmented, CABBAGE provides a strong foundation to advance research and innovation in the AMR space. Additionally, it highlights the need to adopt a standard format for sharing genotype–phenotype data [32], which will facilitate, and aid the automation of, its extension as additional data is generated.

## Supplementary Material

gkag780_Supplemental_File

## Data Availability

The CABBAGE database is freely available via the Antimicrobial Resistance Portal (https://www.ebi.ac.uk/amr), with detailed documentation provided at https://www.ebi.ac.uk/amr/usage/. Access to the ATLAS dataset can be requested from https://amr.vivli.org. The raw supplementary files from publications were processed via custom scripts, available at https://github.com/Leonardini/CABBAGE and https://doi.org/10.5281/zenodo.21133550.

## References

[1] Rose R, Nolan DJ, Ashcraft D et al. Comparing antimicrobial resistant genes and phenotypes across multiple sequencing platforms and assays for *Enterobacterales* clinical isolates. BMC Microbiol. 2023;23:225. 10.1186/s12866-023-02975-x. 37596530 PMC10436404

[2] Rebelo AR, Bortolaia V, Leekitcharoenphon P et al. One DAy in Denmark: comparison of phenotypic and genotypic antimicrobial susceptibility testing in bacterial isolates from clinical settings. Front Microbiol. 2022;13:804627. 10.3389/fmicb.2022.804627. 35756053 PMC9226621

[3] Domanovich-Asor T, Motro Y, Khalfin B et al. Genomic analysis of antimicrobial resistance genotype-to-phenotype agreement in *Helicobacter pylori*. Microorganisms. 2021;9:2. 10.3390/microorganisms9010002 PMC7822022 33374988

[4] Tacconelli E, Carrara E, Savoldi A et al. Discovery, research, and development of new antibiotics: the WHO priority list of antibiotic-resistant bacteria and tuberculosis. Lancet Infect Dis. 2018;18:318–27. 10.1016/S1473-3099(17)30753-3. 29276051

[5] World Health Organization (WHO) , WHO bacterial priority pathogens list, 2024: bacterial pathogens of public health importance to guide research, development and strategies to prevent and control antimicrobial resistance. Technical Report, 2024; Accessed 11th June 2025.

[6] Bissonnette L, Bergeron MG. Infectious disease management through point-of-care personalized medicine molecular diagnostic technologies. J Pers Med. 2012;2:50–70. 10.3390/jpm2020050. 25562799 PMC4251365

[7] Kaprou GD, Bergšpica I, Alexa EA et al. Rapid methods for antimicrobial resistance diagnostics. Antibiotics. 2021;10:209. 10.3390/antibiotics10020209. 33672677 PMC7924329

[8] Holt KE, Carey ME, Chandler C et al. Tools and challenges in the use of routine clinical data for antimicrobial resistance surveillance. npj Antimicrob Resist. 2025;3:37. 10.1038/s44259-025-00105-3. 40346194 PMC12064641

[9] Ajulo S, Awosile B. Global antimicrobial resistance and use surveillance system (GLASS 2022): Investigating the relationship between antimicrobial resistance and antimicrobial consumption data across the participating countries. PLoS One. 2024;19:e0297921. 10.1371/journal.pone.0297921. 38315668 PMC10843100

[10] Osei Sekyere J . Next-generation sequencing in infectious-disease diagnostics: economic, regulatory, and clinical pathways to adoption. Microbiol Open. 2025;14:e70104. 10.1002/mbo3.70104. PMC12658282 41305954

[11] Sundermann AJ, Rosa R, Harris PNA et al. Pathogen genomics in healthcare: overcoming barriers to proactive surveillance. Antimicrob Agents Chemother. 2024;69:e01479–24. 10.1128/aac.01479-24. 39636107 PMC11784250

[12] Aanensen DM, Okeke IN, Donado-Godoy P et al. The genomic surveillance gap: averting the antimicrobial resistance pandemic requires global equity and action. Lancet Infect. Dis. 2026;26:122–23. 41285140 10.1016/S1473-3099(25)00723-6

[13] Antonopoulos DA, Assaf R, Aziz RK et al. PATRIC as a unique resource for studying antimicrobial resistance. Brief Bioinf. 2019;20:1094–1102. 10.1093/bib/bbx083. PMC6781570 28968762

[14] Jolley K, Bray J, Maiden M. Open-access bacterial population genomics: bIGSdb software, the PubMLST.org website and their applications [version 1; peer review: 2 approved]. Wellcome Open Res. 2018;3:124. 10.12688/wellcomeopenres.14826.1. 30345391 PMC6192448

[15] Argimón S, Abudahab K, Goater RJE et al. Microreact: visualizing and sharing data for genomic epidemiology and phylogeography. Microb Genomics. 2016;2:e000093. 10.1099/mgen.0.000093. PMC5320705 28348833

[16] Centers for Disease Control and Prevention (CDC) , Centers for Disease Control and Prevention (U.S.), Antibiotic resistance threats in the United States, 2019. Technical Report, 2019.

[17] Karp BE, Tate H, Plumblee JR et al. National antimicrobial resistance monitoring system: two decades of advancing public health through integrated surveillance of antimicrobial resistance. Foodborne Pathog Dis. 2017;14:545–57. 10.1089/fpd.2017.2283. 28792800 PMC5650714

[18] Matamoros S, Hendriksen RS, Pataki BA et al. Accelerating surveillance and research of antimicrobial resistance–an online repository for sharing of antimicrobial susceptibility data associated with whole-genome sequences. Microb Genomics. 2020;6:e000342. 10.1099/mgen.0.000342. PMC7371118 32255760

[19] Berends MS, Luz CF, Friedrich AW et al. AMR: an R Package for working with antimicrobial resistance data. J Stat Software. 2022;104:1–31. 10.18637/jss.v104.i03.

[20] Hunt M, Lima L, Anderson D et al. AllTheBacteria—all bacterial genomes assembled, available, and searchable. bioRxiv August 28, 2025, pre-print: not peer-reviewed. 10.1101/2024.03.08.584059.

[21] Chklovski A, Parks DH, Woodcroft BJ et al. CheckM2: a rapid, scalable and accurate tool for assessing microbial genome quality using machine learning. Nat. Methods. 2023;20:1203–12. 10.1038/s41592-023-01940-w. 37500759

[22] Gurbich TA, Beracochea M, De Silva NH et al. mettannotator: a comprehensive and scalable Nextflow annotation pipeline for prokaryotic assemblies. Bioinformatics. 2025;41:btaf037. 10.1093/bioinformatics/btaf037. 39854281 PMC11842068

[23] Feldgarden M, Brover V, Gonzalez-Escalona N et al. AMRFinderPlus and the Reference Gene Catalog facilitate examination of the genomic links among antimicrobial resistance, stress response, and virulence. Sci Rep. 2021;11:12728. 10.1038/s41598-021-91456-0. 34135355 PMC8208984

[24] Sumanth G, Gerardo A-U, Paul T et al. Antimicrobial resistance surveillance in low- and middle-income countries: progress and challenges in eight South Asian and Southeast Asian countries. Clin Microbiol Rev. 2020;33:e00048–19. 10.1128/cmr.00048-19.. 32522747 PMC7289787

[25] Iskandar K, Molinier L, Hallit S et al. Surveillance of antimicrobial resistance in low- and middle-income countries: a scattered picture. Antimicrob Resist Infect Control. 2021;10:63. 10.1186/s13756-021-00931-w. 33789754 PMC8011122

[26] Ngabonziza JCS, Rigouts L, Torrea G et al. Multidrug-resistant tuberculosis control in Rwanda overcomes a successful clone that causes most disease over a quarter century. J Clin Tuberc Other Mycobact Dis. 2022;27:100299. 10.1016/j.jctube.2022.100299. 35146133 PMC8802117

[27] Raynaud M, Goutaudier V, Louis K et al. Impact of the COVID-19 pandemic on publication dynamics and non-COVID-19 research production. BMC Med Res Methodol. 2021;21:255. 10.1186/s12874-021-01404-9. 34809561 PMC8607966

[28] Tomczyk S, Taylor A, Brown A et al. Impact of the COVID-19 pandemic on the surveillance, prevention and control of antimicrobial resistance: a global survey. J Antimicrob Chemother. 2021;76:3045–58. 10.1093/jac/dkab300. 34473285 PMC8499888

[29] Rodríguez-Baño J, Rossolini GM, Schultsz C et al. Key considerations on the potential impacts of the COVID-19 pandemic on antimicrobial resistance research and surveillance. Trans R Soc Trop Med Hyg. 2021;115:1122–29. 10.1093/trstmh/trab048. 33772597 PMC8083707

[30] Stotzem N, Guntoro F, Chindelevitch L. BenchmarkDR: a modular and expandable benchmarking pipeline for machine learning based antimicrobial resistance prediction. Machine Learning for Microbial Genomics (MLMG) 2022. https://mlmg2022.github.io/assets/papers/Stotzem.pdf (23 September 2022, date last accessed).

[31] Walter KS, Colijn C, Cohen T et al. Genomic variant-identification methods may alter *Mycobacterium tuberculosis* transmission inferences. Microb Genomics. 2020;6:mgen000418. 10.1099/mgen.0.000418. PMC7641424 32735210

[32] Chindelevitch L, van Dongen M, Graz H et al. Ten simple rules for the sharing of bacterial genotype–phenotype data on antimicrobial resistance. PLoS Comput Biol. 2023;19:e1011129. 10.1371/journal.pcbi.1011129. 37347768 PMC10286994

