# Supplementary material for: A comprehensive AMR genotype–phenotype database (CABBAGE)
Source: Nucleic Acids Res. 2026 Aug 24;54(16):gkag780. doi: 10.1093/nar/gkag780 (PMC13501133; doi:10.1093/nar/gkag780)
Supplement: Supplementary file 1 [file gkag780_supplemental_file.pdf]

S1 SUPPLEMENTARY METHODS

S1.1 Literature review and data extraction

A literature review was carried out, with the initial aim of collecting publicly available datasets containing at least 100 sequenced isolates of bacteria on the WHO's Priority Pathogens List, with antimicrobial resistance phenotypes available for at least one drug. The process recommended by Bramer *et al.* [1] was followed, with the use of the Embase database as the primary source of information. During the refinement process, the minimum sample size for *M. tuberculosis* was increased to 200 due to the large number of available datasets, and decreased to 50 for less commonly represented bacterial pathogens.

A total of six search terms were used, as summarised in Supplementary Table S1. Where a search term generalises a previous one, we assumed that all previous hits would be included, and thus compute and report those statistics only on the new hits. All search terms were restricted to English-language papers, and this included papers from 2010 until the date of the search (July 2022). The search identified 228 unique papers among the 4,325 considered, for an overall precision of 5%. In total, 232 datasets were collected, with a dataset defined as a collection of isolates belonging to a single species, as some of the papers included more than one priority pathogen bacterial species. A further non-systematically identified sample of 28 papers was added, bringing the total number of papers to 256.

During the literature review, papers were excluded if they fell into certain categories. The main categories of excluded results were as follows:

- off-topic papers, such as papers on viruses, fungi, malaria or cancer;
- review papers, position papers, and conference abstracts;
- papers whose sample size was too small (e.g. case studies);
- epidemiology papers (e.g. prevalence papers);
- laboratory or functional genomics papers;
- antibiotic, phage, or antimicrobial peptide identification or design papers;
- studies doing selective sequencing (e.g. known resistance-associated genes).

A number of papers had their data sourced from previously published studies. Any such paper was included provided that it explicitly made the aggregated genotype and phenotype data available, rather

than merely referencing the previous studies; otherwise, the paper was not included, while the referenced previous studies were individually considered for inclusion if they met all the search criteria.

Among the papers identified through the search, eight bacterial pathogens were represented only once, namely, *Escherichia albertii*, *Enterobacter cloacae*, *Enterococcus faecalis*, *Haemophilus influenzae*, *Neisseria meningitidis*, *Serratia marcescens*, *Staphylococcus pseudintermedius*, and *Vibrio cholerae*. Of those, only *H. influenzae* and *S. marcescens* are WHO Priority Pathogens, although several others have appeared on the US CDC [2] or India's Priority Pathogen Lists [3]. The six non-priority pathogens were excluded from analysis, leaving 248 datasets from 224 papers.

For each dataset, an attempt was made to extract the genotype accession numbers as well as the phenotype tables, in the first instance from the Supplementary Materials of the paper. If this was not successful, the full text of the paper was analysed to identify included accession numbers (by textual search for the terms "access", "avail", and "depos") and phenotype tables (by visual inspection of the tables and figures). As a result of one of the types of data (either genotypes or phenotypes) being unavailable, the original set of papers was narrowed down to 127 datasets from 107 papers.

Papers reporting the genotypic prediction of resistance and its concordance with phenotypic resistance were excluded if they did not explicitly specify the discrepant isolates, or if they did but only provided the concordant phenotypes implicitly as the results of a prediction tool, since such tools tend to be updated over time and may yield predictions that disagree with their original ones. The sample size was also reduced for several datasets, as not all strains under study were publicly available, and 14 were then excluded due to the remaining sample size being too small (below 100), resulting in 113 datasets from 94 papers. As some datasets used identical sources for both their genotypic and phenotypic data, they were deduplicated, leaving 110 unique datasets from 91 papers. A flow chart illustrating the literature review process and the number of papers excluded at each stage of processing is displayed in Supplementary Figure S1.

S1.2 Initial data processing

Several papers only had their data available in formats that are challenging to process via automated scripts, such as PDF files containing tables or PNG figures. In this instance, the paper's corresponding author was contacted with a request to provide the data in a machine-readable format. The authors

were contacted a second time after 5 working days, and if no reply was received after a further 5 working days, the data was considered unusable for data collection and removed from further consideration. Seven out of eleven corresponding authors complied with the request within 10 working days. Four failed to respond, resulting in an attempt to "manually" convert 3 PDF files into CSV files. One dataset whose author did not respond to the request was excluded due to only having phenotype data in the format of a phylogenetic tree with no labels. No contact was made with authors of papers that did not include both genotype and phenotype data, even if these were stated to be available upon request.

For each dataset with publicly available data, we extracted tables containing the phenotypes as well as the accession numbers for the genotypes, with a preference for raw sequencing data when it was available; we used assembled contigs when no raw sequencing data was available, but did not include any papers that only provided access to variant calls as these may be difficult to reproduce. The hyperlink corresponding to each table and study was recorded. When the article was freely available in PMC we used its PMC version as the source of the data; however, some of the articles were behind a paywall and the links we provide to the data in those may not be accessible at all institutions, hindering the open access of the data and the reproducibility of our results.

The tables were first standardised into a CSV format (comma-separated rather than semicolon-separated) manually due to the variability of formats. For Excel files, rows before the header and after the final row were removed; sometimes this necessitated the removal of columns (for instance, if the header was on two levels, with the first level providing the drug name and the second level stating if the reported result was experimentally measured or computationally predicted, the computational predictions had to be removed to ensure lossless conversion into a CSV format). For Excel files with multiple sheets, only the relevant tabs were converted, and when multiple datasets were provided as separate sheets, each sheet was treated separately and then merged if appropriate (e.g. a training dataset and a testing dataset could be merged into a single overall dataset if their formats were compatible).

For those genotypes available only as an INSDC project, the RunSelector tool (NCBI) was used to extract the correspondence between internal IDs/sample names and accession numbers. For those papers where the genotype and the phenotype information were provided as separate tables (including those with genotypes available via an INSDC project), the tables were merged using an inner join (i.e. keeping only those isolates with both phenotype and genotype information available), using manually determined identifier columns in non-trivial cases, except when the manuscript clearly specified a default phenotype

that was then assumed for all samples present in the genotypic table but absent in the phenotypic table. One additional dataset was excluded due to having no identifiers usable for matching and no reply to a request to its authors for a dictionary to establish the correspondence. This left a total of 108 datasets from 89 papers.

S1.3 Database analysis

The following lists the URL's for databases that were assessed for integration into CABBAGE:

- Bacterial and Viral Bioinformatics Resource Center (BV-BRC), at https://www.bv-brc.org;
- EnteroBase, at http://enterobase.warwick.ac.uk;
- MGTdb, at http://mgtdb.unsw.edu.au;
- Microreact, at http://microreact.org;
- National Antimicrobial Resistance Monitoring System for Enteric Bacteria (NARMS), at http://www.cdc.gov/narms/index.html;
- National Database of Antibiotic Resistant Organisms (NDARO), at http://ncbi.nlm.nih.gov/pathogens;
- PathogenWatch, at https://pathogen.watch;
- PubMLST, at http://pubmlst.org;
- European COMPARE Consortium (AMR Data Hub), at https://www.ebi.ac.uk/ena/datahubs/active-datahubs/datahub/dcc_schubert;
- NCBI antibiograms, at https://www.ncbi.nlm.nih.gov;
- CDC, at https://wwwn.cdc.gov/arisolatebank/.

Unfortunately, EnteroBase does not contain any AMR phenotype information except for a small number of *Helicobacter pylori* isolates, while MGTdb only contains such information for *Bordetella pertussis*, where it is computationally rather than experimentally predicted. Consequently, these two databases (EnteroBase and MGTdb) were excluded. We thus focus our initial efforts on the remaining 9 databases: BV-BRC, NARMS, NDARO, PathogenWatch, pubMLST, the European COMPARE Consortium, NCBI, Microreact, and the CDC.

S1.3.1 BV-BRC

For the BV-BRC database (formerly known as PATRIC) we followed the recommendation of Van Oeffelen *et al.* [4] and downloaded the entire contents of the database from the links at https://docs.patricbrc.org/user_guides/ftp.html. Because many phenotype entries lacked a genomic identifier and included only a sample name, the corresponding sequencing run IDs were retrieved either from the source publications (2,362 isolates; PubMed IDs 32025709, 30373719, 31537784, 29133554, and 34485958) or from the BioSample database, when the search returned a single matching BioSample ID whose species matched that of the corresponding BV-BRC entry (3,676 isolates). Filtering the BV-BRC entries to species on the WHO Priority Pathogen list resulted in a total of 83,267 unique isolates. The final number of entries sourced from BV-BRC after processing are summarised in Table S2.

S1.3.2 The European COMPARE Consortium

The European COMPARE Consortium [5] has previously attempted to standardise the reporting of AMR phenotypes, and there are a total of 2,570 isolates (790 *Escherichia coli* and 1,780 *Salmonella enterica*), together with antibiograms, included in its AMR Data Hub. We include this data from the AMR Data Hub in the current analysis because, although much of it is also found in other sources, the formatting of the antibiogram data allowed for straightforward integration into existing workflows. However, we note that for one of the 12 studies included in the AMR Data Hub (PRJDB7087), each of the isolates has a repeated value for three antibiotics (aztreonam, cefotaxime, and cefotiam), so these antibiotics are excluded from our analysis in this study.

S1.3.3 Microreact

Since Microreact projects are not explicitly linked to from its homepage, except for the Showcase selection [6], we followed each of the five showcased datasets focused on bacterial pathogens in the WHO Priority Pathogens List to its original publication. The first was already included in our search [7]; another [8], although not identified in our search, lacked antimicrobial resistance data in the Microreact table, which was instead extracted from the original publication. The remaining three [9, 10, 11] either did not contain any antimicrobial resistance data, or had fewer than 100 strains, and thus were not used. We also examined the 21 Microreact collections at https://www.microbiologyresearch.org/content/microreact to identify any that had additional data meeting our search criteria, and found five matching datasets, one of which was included in our original search.

S1.3.4 NARMS

NARMS primarily contains information on foodborne pathogens. We query it for genotyped isolates from 1999 onwards with AST-based phenotypes, excluding *Vibrio* which is not part of our search targets.

S1.3.5 NDARO

NDARO contains information from NCBI on a number of pathogens and their antimicrobial resistance. It can be accessed via FTP at `ftp://ftp.ncbi.nlm.nih.gov/pathogen`. Each genus or species of pathogen is collected into its own folder, and the AMR subfolder contained in each of those provides a file that specifies the combination of accession number and AST profile, along with many other metadata fields. We downloaded these files for each of the bacterial pathogens on the WHO Pathogen Priority list.

S1.3.6 PathogenWatch

The PathogenWatch database is organised into 89 collections of isolates. Of those, 44 have fewer than 100 isolates, and are therefore excluded from our analysis. Of the remaining 45 collections, a further 3 contain pathogens not targeted by our search, and one (for the EuroGASP 2013 collection) contains no identifiers that could lead to accession numbers. The 41 remaining collections were processed by downloading the metadata and the AMR profile in csv format, merging them using a custom script and filtering out any isolates which only had the genotype or the phenotype available, but not both, as well as querying EBI for obtaining run accession numbers from the sample accession numbers when needed. Finally, the files were processed to remove duplications.

S1.3.7 pubMLST

Since pubMLST is primarily focused on providing multilocus sequence type information, the incorporation of resistance profiles seems to be a secondary goal. For this reason, most of the pathogens of interest to us did not contain enough isolates with whole genomes as well as resistance profiles to be selected, with the following exceptions (Table S2), found by querying the genome collection for each relevant species with the ‘Field NOT null’ search specification, where Field was ‘resistance profile’, ‘drug resistance’, or antibiotic names combined via OR.

S1.4 Metadata extraction

To improve the completeness of the database, relevant metadata was extracted directly from the manuscripts and supplementary materials of the 108 datasets collected from 89 papers during the literature review. This metadata was recorded in a metadata CSV file, separate to the CABBAGE dataset, with the aim

of incorporating metadata present in the manuscript or supplementary materials, but absent in the original datafile. Each manuscript and its associated supplementary materials were manually examined and, where relevant information was identified, it was highlighted within the text and recorded within fields based on those used by NCBI BioSample Antibiograms. These fields include 'Laboratory typing method', 'Laboratory typing platform', 'Testing standard', 'Testing standard documentation', 'Vendor' and 'Laboratory typing method version or reagent'. In addition to these fields, we defined three additional fields: 'Temperature (°C)', 'Incubation time (hrs)' and 'Conditions'. However, due to the sparsity of information available for these three fields (the temperature, incubation time and conditions were only specified in 6, 7 and 2 papers respectively), this information was not incorporated into the final database.

For a number of papers, the methods and/or testing standards were provided for specific antibiotics, and so these were listed alongside the corresponding antibiotic in the field 'Laboratory typing platform - antibiotic specific' or 'Testing standard - antibiotic specific'. Where different methods were applied to different sets of isolates within a paper, the entry was split, and this was indicated in the 'Duplicate' field with a 'yes' or 'no' entry. An additional 'Notes' field includes any information relevant to the metadata, for instance the specific breakpoints that were used, if different methods were used for different subsets of isolates, and if these subsets were ambiguous. Note that fields such as 'Notes' and 'Duplicate' are not included in CABBAGE, as their purpose was to provide additional information required for the decision-making process to incorporate metadata into CABBAGE.

S1.5 Data processing and standardisation

Bacterial pathogens not included in the WHO Priority Pathogen list were removed from further analysis, and several species were renamed for consistency as follows: *Salmonella* Typhimurium, *Salmonella* Senftenberg and *Salmonella enterica* subsp. *enterica* serovar Kentucky to *Salmonella enterica*; *Enterobacter cloacae* complex and *Enterobacter cloacae* group to *Enterobacter cloacae*; and finally *Providencia* species to *Providencia stuartii*.

It was noted that a number of PathogenWatch datasets were derived from the 'AMR profile', consisting of phenotypic resistance predictions based on antibiotic resistance genes. As a result, these datasets were removed from further analysis, reducing the number of datasets from PathogenWatch from 41 to 21. Two additional datasets [12, 13] derived from the literature search were excluded from further analysis as an excessive number of assumptions would otherwise be required to reliably interpret the phenotypic data.

Antibiotic abbreviations were derived from the antibiotic name provided in the original dataset, if available, based on the names and corresponding abbreviations used in the AMR package [14]. To ensure accuracy in the assignment of antibiotic names in cases where only the abbreviation is provided in the original dataset, the manuscript and supplementary materials were referred to. In some cases a single antibiotic abbreviation corresponds to multiple antibiotic names across different data sources, for instance, the abbreviation CFX was used to denote either cefuroxime, cefixime or ceftriaxone. The abbreviations were therefore converted into CXM, CFM and CRO respectively. Additionally, several antibiotic names were standardised to align with those given in the AMR package, for example co-amoxyclav was changed to amoxicillin-clavulanic acid and co-trimoxazole to trimethoprim-sulfamethoxazole. Entries with only the antibiotic class recorded were not incorporated into CABBAGE if the specific antibiotic could not be determined from the original paper or Supplementary Materials.

In several instances, commonly used antibiotic abbreviations in the source materials conflicted with the abbreviation scheme used here. This was particularly apparent for tetracycline and streptomycin that frequently have the abbreviations TET and STR respectively, referring to tetroxoprim and streptoduocin in the AMR package. These discrepancies were resolved through manual verification against the antibiotic names provided in the source materials.

A variety of phenotypes are recorded in CABBAGE, and they are as follows: susceptible, resistant, intermediate, susceptible-dose dependent and non-susceptible. Where possible, phenotypes were standardised, for example, 'S' and 'sensitive' were changed to 'susceptible' and 'R' to 'resistant'. Several datasets represent phenotypes numerically, for instance, 0 may represent susceptible and 1 resistant. In these cases, the original source was consulted to convert these numbers to their corresponding R/I/S classification.

The testing standard refers to internationally recognised guidelines, such as those set out by CLSI, which define the interpretive criteria (breakpoints) for classifying antibiotic susceptibility. For consistency, the full organisation names were converted into their corresponding abbreviation, for example, 'Clinical and Laboratory Standards Institute' was changed to CLSI. Ambiguous testing standards were omitted, for instance if multiple standards were reported for a single entry.

Further steps include the standardisation of the host information to either '*Homo sapiens*' or 'other (non-clinical isolate)', with the latter encompassing all non-human entries such as animals, the environment, and food. The isolation countries were standardised using ISO 3166-1 alpha-3 codes (ISO3), which are

three-letter country codes defined by the International Organisation for Standardisation (ISO). Several entries could not be converted into an ISO3 code because they do not correspond to a single country, for example, 'Caribbean'. Several country names were also updated to reflect current official naming conventions, for instance, Ivory Coast is officially designated as Côte d'Ivoire under the ISO standard, and corresponds to the ISO3 code CIV.

S1.6 Data curation

MICs are recorded in various formats, including integers (e.g. 2), floats (e.g 0.5), intervals (e.g. 2,5) and combinations (e.g. 2/38). It should be noted that MICs for antibiotic combinations (such as trimethoprim-sulfamethoxazole) are given as either a single MIC, or a MIC combination listing the concentrations of each component separately, depending on the format given in the original datafile. Several MICs were excluded due to probable reporting errors. This includes unusually large values inconsistent with standard doubling dilutions (e.g. 42,023 and 1,000,000), zero or null values (0.0 or 0) reported alongside 'broth dilution' as the laboratory typing method, incomplete MICs (e.g. .016), incomplete antibiotic combinations (e.g. 0.06/, /64, /128), and values with other formatting anomalies such as trailing full stops (e.g. 0.5/9.). Where possible, the measurement sign associated with each MIC was collected directly from the dataset, or otherwise recorded as '=='. MIC intervals represent the range of MIC values that lie between two defined thresholds, and so are assigned the measurement sign '>,<'. Several categorical phenotypes were removed from the database (decreased susceptibility, high level resistance, SYN-R and SYN-S), while the following categorical phenotypes were retained : susceptible, resistant, intermediate, non-susceptible, and susceptible-dose dependent.

Two measurement units are reported in CABBAGE (mg/L and mm), however if no measurement unit is provided in the original dataset, it was derived from the AST method where possible. For example, if the laboratory typing method is broth dilution, E-test or agar dilution, the measurement unit is recorded as 'mg/L', whereas 'mm' is used in the case of disk diffusion. Mismatches between the measurement unit and laboratory typing method were identified, such as entries with 'mg/L' recorded alongside 'disk diffusion'. One dataset [15] provides the units 'mg/ml' for *Mycobacterium tuberculosis* and ethionamide, however this was presumed to be an error due to the numerical values involved and changed to 'mg/L'.

The laboratory typing method field refers to the AST methodology used to generate the MIC and/or resistance phenotype, with entries recorded as either broth dilution, E-test, agar dilution or disk diffusion. It should be noted that within CABBAGE, the term 'broth dilution' is used to represent both broth

microdilution and macrodilution. Where entries lacked a laboratory typing method, the metadata file (as described in Supplementary section S1.4) was consulted and the method was only recorded if it was unambiguous, for example, if a single method was used across all isolates within the study. If entries lacked a laboratory typing method that could not be resolved via this strategy, it was derived from the laboratory typing platform where possible, for example, if the laboratory typing platform was recorded as Vitek, Sensititre, BD Phoenix, Microscan or MGIT900, the method was recorded as broth dilution.

Entries for age were rounded to the nearest whole number, and those that appeared to be a reporting error, such as 999, were removed. For consistency between entries with different date formats, only the collection year was recorded rather than the full date. The only collection year to be removed was '1800', as this was believed to be a reporting error.

AMR associated publications correspond to specific publications indexed in PubMed, enabling users of CABBAGE to refer to the original data source. However, database-derived PubMed IDs should be interpreted with caution, as they don't consistently correspond to the correct publications. Several entries are associated with multiple PubMed IDs and are recorded as such, whilst other entries have no associated PubMed ID, for example if the genome and antibiogram data were deposited directly to NCBI without a corresponding publication.

The final stage of curation involved removing duplicate entries by grouping together records sharing the same BioSample ID and antibiotic name. This step also helped identify entries with conflicting phenotypes. A conflicting phenotype was defined as differing measurement values obtained using the same AST method, or - when no measurement was available - differing SIR phenotypes derived from the same method. Groups containing conflicts were discarded, except when the conflicting entries originated from the same source, in which case within-study replicates were retained. Groups without conflicts were further deduplicated as follows: entries were first ranked by the number of filled phenotype fields (see Figure 2), giving highest priority to measurement values (for example, an entry with only a MIC value was preferred over one containing only SIR phenotype, AST method, and platform). The entry with the highest score was selected as the representative of the group, except when conflicting entries originated from different AST methods, in which case both entries were retained as representatives. Finally, a majority-rule consensus was applied to metadata fields, and the resulting consensus values were combined with the phenotype fields of the representative entry or entries.

To address the use of outdated interpretive breakpoints to infer phenotypes, we incorporated updated phenotypes, applying the current breakpoint criteria from both CLSI and EUCAST, inferred using the AMR package v3.0.0 [14]. Epidemiological cutoff values (ECOFFs) were used when EUCAST breakpoints were unavailable, with their use indicated in the 'used ECOFF' field as a yes or no entry. Breakpoints for *Salmonella enterica subsp. enterica* were used to determine updated phenotypes for *Salmonella enterica*.

S1.7 Data analysis

The origin of CABBAGE data and the overlap between data sources was visualised using ComplexUpset (version 1.3.5) in R. GeoPandas (version 1.1.1) in Python was used to map the global distribution of entries and their resistance phenotypes, restricted to countries with at least 500 isolates linked to a resistance phenotype for the latter. The comparative analysis of CABBAGE with ATLAS was carried out on CABBAGE data that was filtered to entries with a collection year between 2004-2023 to reflect the range of collection dates in ATLAS. An analysis of CABBAGE data that was not filtered by collection year was also performed. The CABBAGE phenotypes derived from the original data source were used for this analysis, rather than the updated phenotypes. A binomial generalised linear model (logit link) in Python using statsmodels was used to assess bias towards resistance in either dataset. Fisher's exact test was applied to identify specific pathogen-antibiotic pairs that showed significant bias towards susceptibility or resistance.

S1.8 AMR portal data preparation and execution

Unassembled bacteria genomes were generated using Shovill (v1.1.0) with contigs screened against the T2T CHM13 version 2 assembly (GCA 009914755.4) with MUMmer (v4.0.0rc1) to remove human contamination. As part of the mettannoator annotation (v1.5.0), AMRFinderPlus (v4.0.23) using database version 4.0 2025-07-16.1 was used.

Both phenotype and genotype records were transformed into CSV files using a set of custom python scripts (https://github.com/Ensembl/amr_genotypes), normalised and then converted into a final set of parquet and CSV data files. Antibiotic agents were converted to Antibiotic Resistance Ontology (ARO) terms using EMBL-EBIs Ontology Lookup Service [16] [17]. Fields such as measurement, resistance phenotype and species information were additionally harmonised. The AMR portal (https://github.com/Ensembl/amr-portal/) ETL suite provides a configuration file format to describe data sets, create composite representations and annotate as URLs or similar. After an ETL run a single

DuckDB file is generated representing the portal data set and contains all configuration needed. The portal was built using a Python FastAPI backend which creates SQL statements to filter each data set accordingly. The front-end is a single page application using the lit framework.

S1.9 Supplementary Results

The inclusion of PubMed IDs and database names in CABBAGE entries allow, when available, users to refer to the original data source. however, this information isn't available for every entry, and where the PubMed ID is recorded, the majority of isolates are associated with a single ID, while others are associated with up to eight (Figure S4).

The number of entries available for each antibiotic is pathogen specific. Consequently, some antibiotics have been tested more frequently against certain pathogens than others, as illustrated in Supplementary Figure S5. For instance, the most frequently tested antibiotics for *M.tuberculosis* are rifampin (12.4%) and isoniazid (12.2%), consistent with their role as first-line treatments. In contrast, amongst the 91 most common antibiotics in CABBAGE (>1,000 entries), eight (erythromycin, fusidic acid, gentamicin, methicillin, mupirocin, penicillin, tetracycline and vancomycin) have only a single AST result each, while 62 have no data for *M.tuberculosis*. Amongst the antibiotics tested against the widest range of pathogens are ciprofloxacin (54/58 pathogens), gentamicin (53/58), meropenem (52/58) and ceftriaxone (52/58), highlighting their use as broad-spectrum antimicrobials. Conversely, 43 antibiotics were only tested against a single pathogen each.

The availability of AST data (either MIC, phenotype, or both) also varies between different antibiotics, as shown in Supplementary Figure S9. For instance, ciprofloxacin is the most frequently tested antimicrobial (>96,000 entries), while dicloxacillin, ceftizoxime and mecillinam have the least data availability, each represented by only a single AST result. Broad-spectrum antibiotics and/or those that are commonly used in clinical settings are, as might be expected, the most frequently represented in CABBAGE, while there is less available data for narrow-spectrum and rarely used antimicrobials.

S1.10 Supplementary Tables and Figures

Search term	Hits	Relevant	Precision
("antibiotic resistance" OR "antibiotic sensitivity" OR "drug resistance" OR "multidrug resistance" OR AMR OR MDR) AND ("genotype phenotype correlation" OR "genotype phenotype prediction") AND ("whole genome sequencing" OR WGS)	84	21	25%
("antibiotic resistance" OR "antibiotic sensitivity" OR "antimicrobial resistance" OR "antimicrobial sensitivity testing" OR "drug resistance" OR "multidrug resistance" OR AMR OR AST OR MDR) AND ("genotype phenotype correlation" OR "genotype phenotype prediction" OR "genotype phenotype association" OR "genome wide association study" OR GWAS) AND ("next generation sequencing" OR "whole genome sequencing" OR NGS OR WGS)	197	32	16%
(antibiotic OR antimicrobial OR drug) AND resistance AND sequencing AND accession	100	6	6.0%
(antibiotic OR antimicrobial OR drug) AND resistance AND sequencing AND prediction	1205	89	7.4%
predict* NOT prediction AND (antibiotic OR antimicrobial OR drug) AND resistance AND sequencing	1760	43	2.4%
sequenc* NOT sequencing AND predict* AND (antibiotic OR antimicrobial OR drug) AND resistance	979	16	1.6%

Table S1: Terms used in the literature search, with a summary of their results

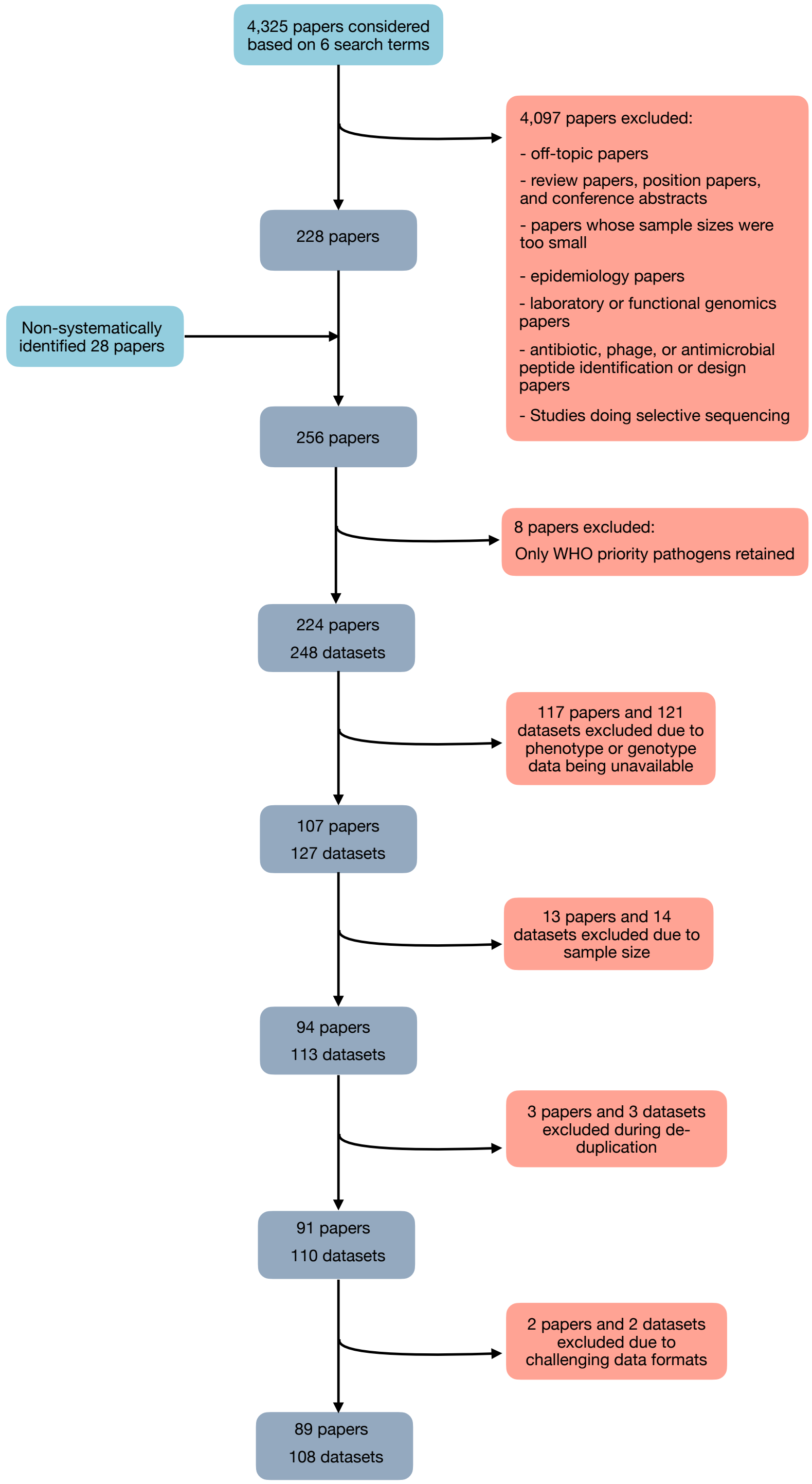

Figure S1: The number of papers and datasets excluded at each stage of the literature review process.

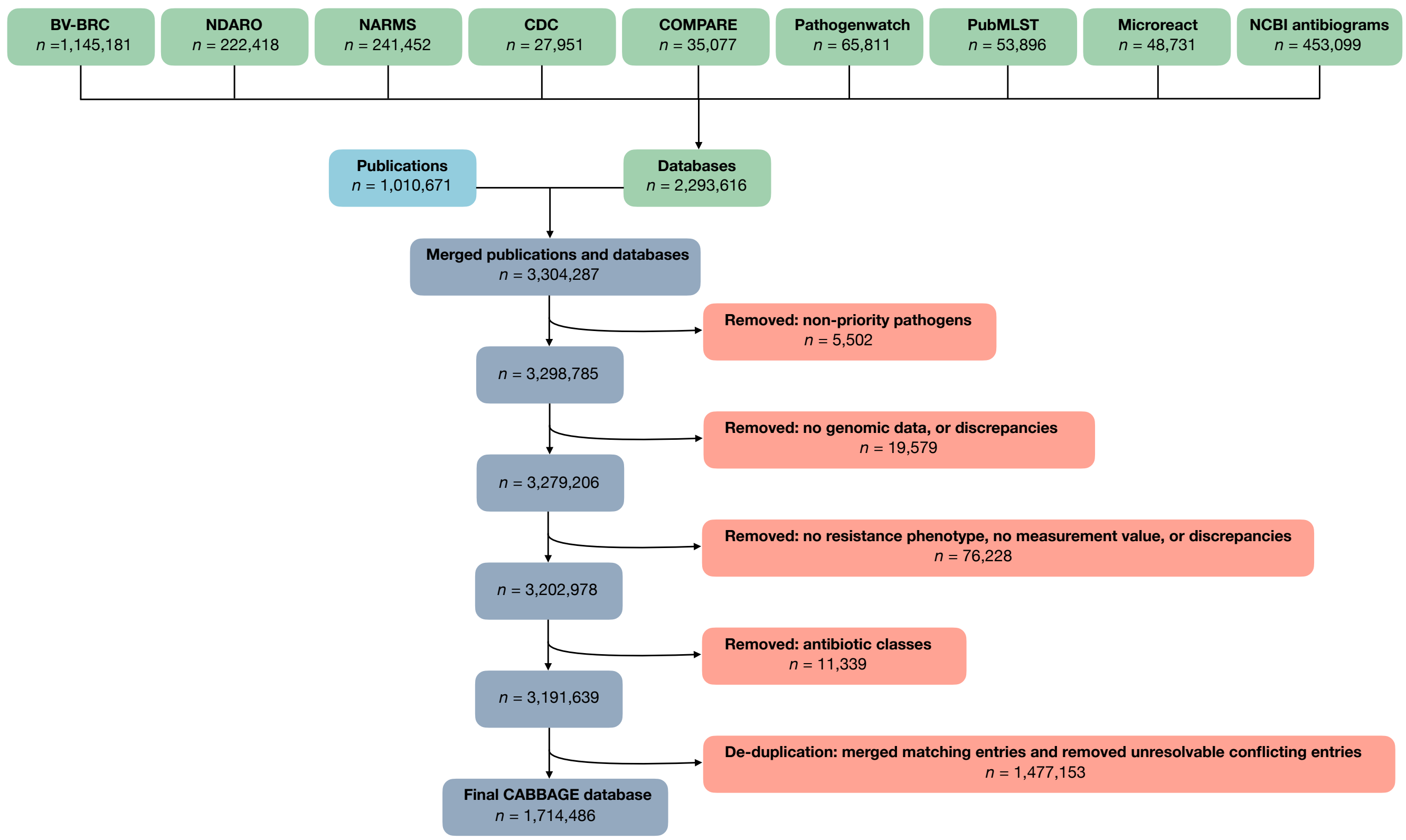

Figure S2: The number of entries removed at each stage of curation and de-duplication.

Database	Pathogen	Number of entries
BV-BRC	*Acinetobacter baumannii*	21,644
	Campylobacter coli & jejuni	8,301
	Clostridioides difficile	3,990
	Enterobacter cloacae & hormaechei	16,400
	Enterococcus faecium	7,557
	Escherichia coli	172,631
	Haemophilus influenzae	705
	Helicobacter pylori	265
	Klebsiella pneumoniae	109,185
	Morganella morganii	1,399
	Mycobacterium tuberculosis	72,085
	Neisseria gonorrhoeae	45,877
	Proteus mirabilis	1,724
	Providencia rettgeri & stuartii	377
	Pseudomonas aeruginosa	9,839
	Salmonella enterica	151,476
	Serratia marcescens	4,154
	Shigella flexneri & sonnei	18,465
	Staphylococcus aureus	32,441
	Streptococcus pneumoniae	78,087
CDC	*Acinetobacter baumannii*	1,106
	Clostridioides difficile	180
	Enterobacter cloacae	1,154
	Enterococcus faecium	83
	Escherichia coli	3,027
	Klebsiella pneumoniae	3,716
	Morganella morganii	76
	Neisseria gonorrhoeae	357
	Proteus mirabilis	262
	Providencia rettgeri & stuartii	151
	Pseudomonas aeruginosa	1,919
	Salmonella enterica	71
	Serratia marcescens	316
	Shigella flexneri & sonnei	241
	Staphylococcus aureus	1,895
COMPARE	*Escherichia coli*	11,214
	Salmonella enterica	21,266
Microreact	*Klebsiella pneumoniae*	4,319
	Streptococcus pneumoniae	39,418
	Salmonella enterica	1,843
NARMS	*Campylobacter coli & jejuni*	24,208
	Salmonella enterica	195,248
	Escherichia coli	10,853
	Shigella sonnei & flexneri	9,561
NDARO	*Acinetobacter baumannii*	10,717
	Campylobacter coli & jejuni	30,487
	Enterobacter cloacae & hormaechei	1,805
	Enterococcus faecium	94
	Escherichia coli	45,750
	Klebsiella pneumoniae	6,714
	Morganella morganii	57
	Neisseria gonorrhoeae	56
	Proteus mirabilis	128
	Providencia rettgeri & stuartii	95
	Pseudomonas aeruginosa	1,681
	Salmonella enterica	114,779
	Serratia marcescens	165
	Shigella sonnei	160
	Staphylococcus aureus	1,533
	Streptococcus pneumoniae	3,506
PathogenWatch	*Neisseria gonorrhoeae*	56,686
	Staphylococcus aureus	5,278
PubMLST	*Haemophilus influenzae*	139
	Staphylococcus aureus	24
	Neisseria gonorrhoeae	45,690
	Neisseria meningitidis	2,659
	Streptococcus pneumoniae	464

Database	Pathogen	Number of entries
NCBI antibiograms	*Escherichia coli*	154,339
	Salmonella enterica	141,579
	Mycobacterium tuberculosis	26,899
	Klebsiella pneumoniae	25,315
	Campylobacter coli & jejuni	35,623
	Acinetobacter baumannii	17,157
	Staphylococcus aureus	12,872
	Pseudomonas aeruginosa	7,002
	Neisseria gonorrhoeae	5,937
	Streptococcus pneumoniae	3,792
	Enterobacter cloacae & hormaechei	4,148
	Serratia marcescens	891
	Shigella sonnei & flexneri	924
	Enterococcus faecium	537
	Proteus mirabilis	533
	Enterobacter sp.	410
	Enterobacter roggenkampii	408
	Clostridioides difficile	373
	Morganella morganii	288
	Enterobacter asburiae	188
	Providencia rettgeri & stuartii	256
	Enterobacter kobei	166
	Enterobacter aerogenes	155
	Enterobacter ludwigii	99
	Enterobacter bugandensis	96
	Proteus vulgaris	70
	Campylobacter lari	63
	Serratia sp.	48
	Serratia liquefaciens	28
	Serratia rubidaea	26
	Proteus terrae	18
	Proteus columbae	16
	Proteus sp.	15
	Shigella boydii	14
	Enterobacter	14
	Enterobacter mori	14
	Serratia fonticola	14
	Helicobacter pylori	6
	Salmonella sp.	4
	Providencia sp.	3
	Enterobacter chengduensis	3

Table S2: The number of entries for each pathogen from each database after processing. Note that these entries may be shared amongst multiple databases.

Database	Number of entries
BV-BRC	764,007
CDC	14,657
COMPARE	32,480
Microreact	45,580
NARMS	240,218
NDARO	217,946
PathogenWatch	61,964
PubMLST	48,976
NCBI antibiograms	440,343

Table S3: The number of entries contributed by each database after processing. Entries may be shared amongst multiple databases.

**Feature**	**Count**
Genomes	170,750
Measurement values	1,299,368
Phenotypes	1,379,999
Genera	20
Antibiotics	165

Table S4: Features of CABBAGE and their corresponding counts. The phenotype count represents the number of phenotypes extracted from the original data source, rather than the updated phenotype (EUCAST or CLSI).

PubMed ID	Laboratory Typing Method
22914622	broth dilution
23299977	broth dilution
24277043	E-test
24462211	agar dilution
25378573	agar dilution
25903077	disk diffusion
26135860	broth dilution
26806258	disk diffusion
26935729	agar dilution
26974227	E-test
27067331	broth dilution
27150362	disk diffusion
27322919	broth dilution
27432602	E-test
28931025	disk diffusion
29017452	broth dilution
29295910	disk diffusion
29377579	agar dilution
29547094	broth dilution
29553335	agar dilution (SPT) and E-test (all other antibiotics)
29617860	broth dilution
29701830	disk diffusion (PEN,TCY) and E-test (all other antibiotics)
29723570	E-test
29882175	E-test
30820563	agar dilution
31005733	broth dilution
31010866	broth dilution
31038449	broth dilution
31220112	E-test
31253105	disk diffusion (AMP, CHL, SXT) and broth dilution (all other antibiotics)
31358980	E-test
31424550	E-test
31488838	agar dilution
31504566	E-test
31530672	broth dilution
31665255	E-test
31941492	broth dilution (COL, DOX,PLB) and disk diffusion (all other antibiotics)
31978353	agar dilution
32010104	disk diffusion
32013864	E-test
32048461	agar dilution
32068837	E-test
32091356	agar dilution
32205351	broth dilution
32422315	disk diffusion
32829411	E-test
32868325	E-test
32977759	E-test
33367806	E-test
34161790	agar dilution
34570642	broth dilution
34693903	broth dilution
34745054	broth dilution
34819373	broth dilution
35156028	broth dilution
35178041	broth dilution
35260883	broth dilution
35308374	agar dilution
35378228	broth dilution
35651495	broth dilution
35731173	broth dilution
35880764	agar dilution
35916524	broth dilution
36165686	broth dilution
36208202	agar dilution
36473954	broth dilution
36801013	broth dilution
37316492	broth dilution
37327220	E-test (CIP, AZM) and disk diffusion (all other antibiotics)
37549252	disk diffusion
38052776	broth dilution

Table S5: PubMed ID's of datasets obtained from BV-BRC and the corresponding laboratory typing methods, determined by referring to the original manuscript and Supplementary Materials.

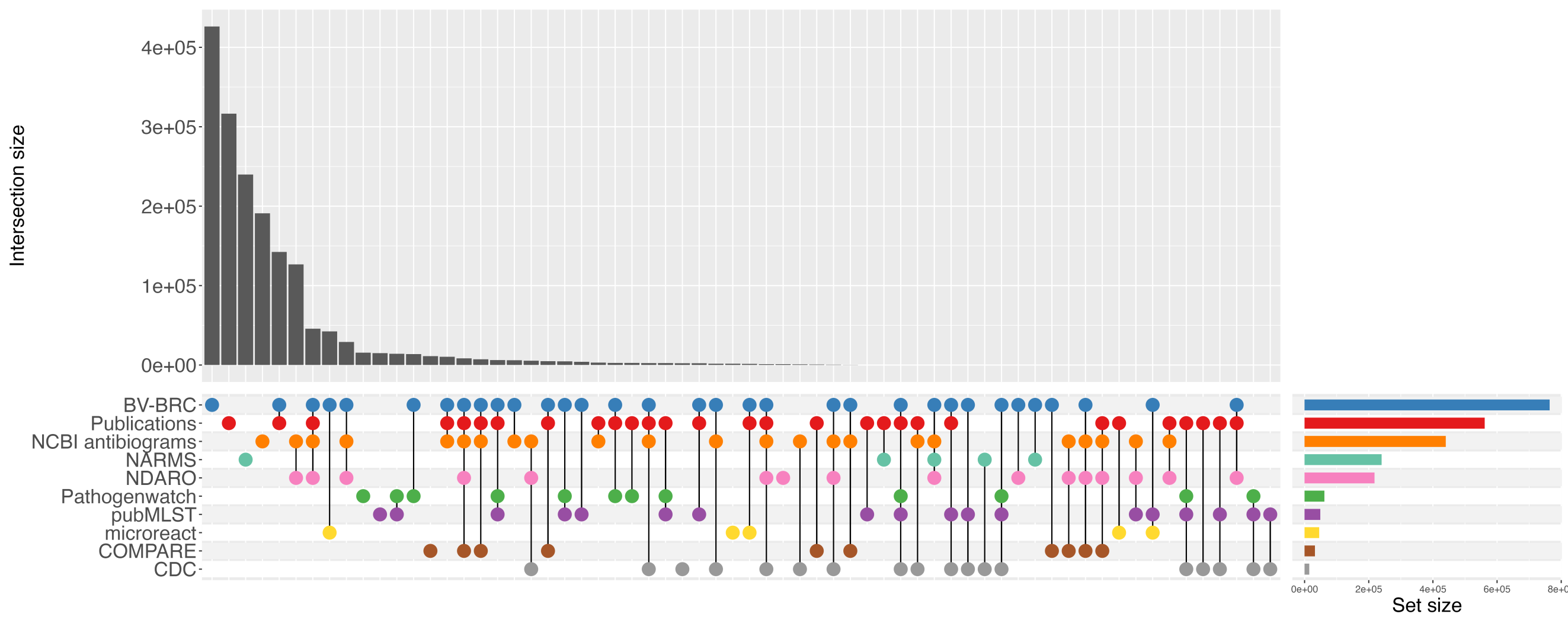

Figure S3: An UpSet plot illustrating the origin of data within the CABBAGE dataset, and the overlap between those sources. Data was sourced from publications, BV-BRC, NCBI antibiograms, NARMS, NDARO, Pathogenwatch, PubMLST, Microreact, COMPARE and the CDC.

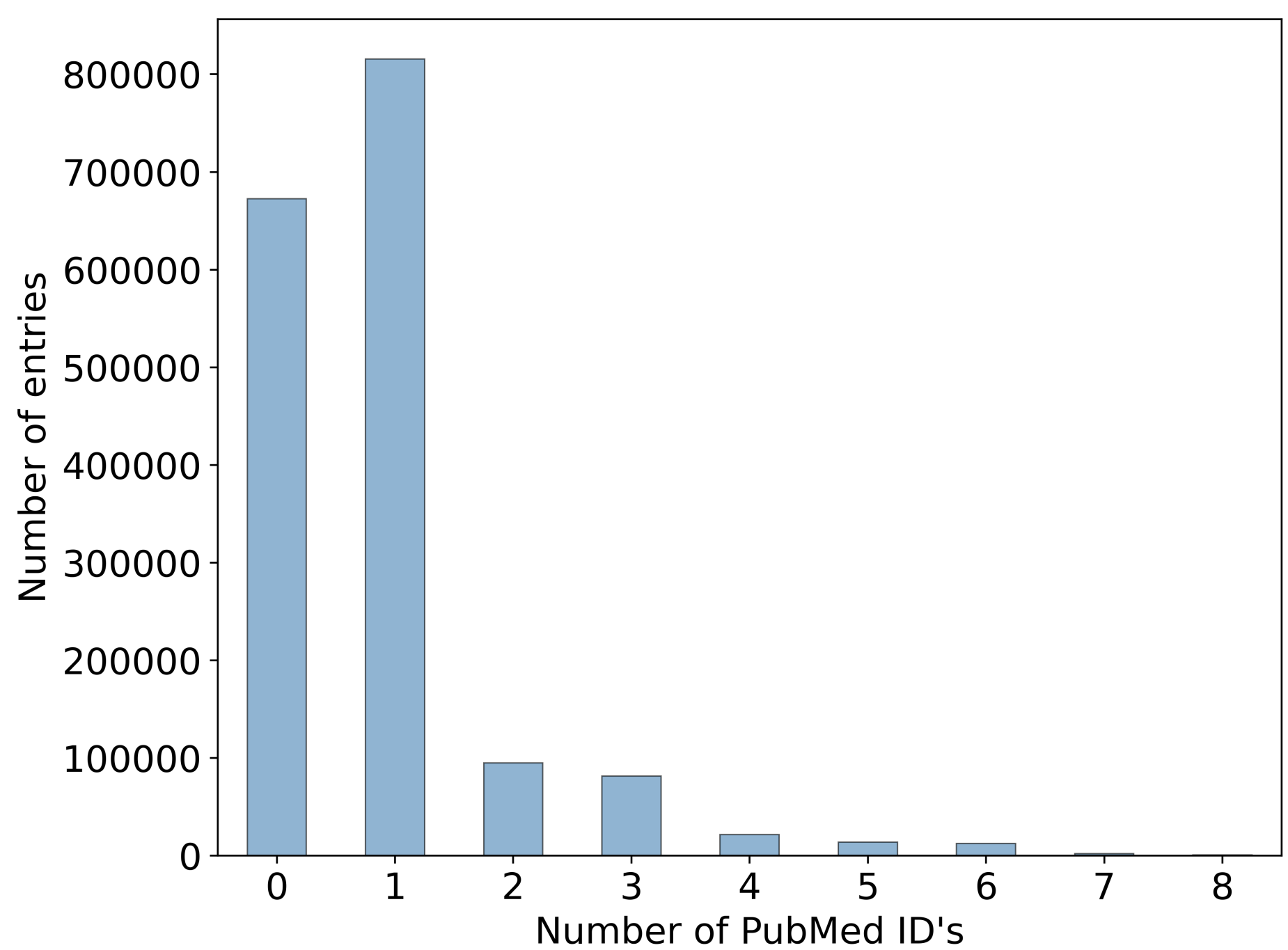

Figure S4: The number of entries associated with multiple, a single, or no PubMed ID.

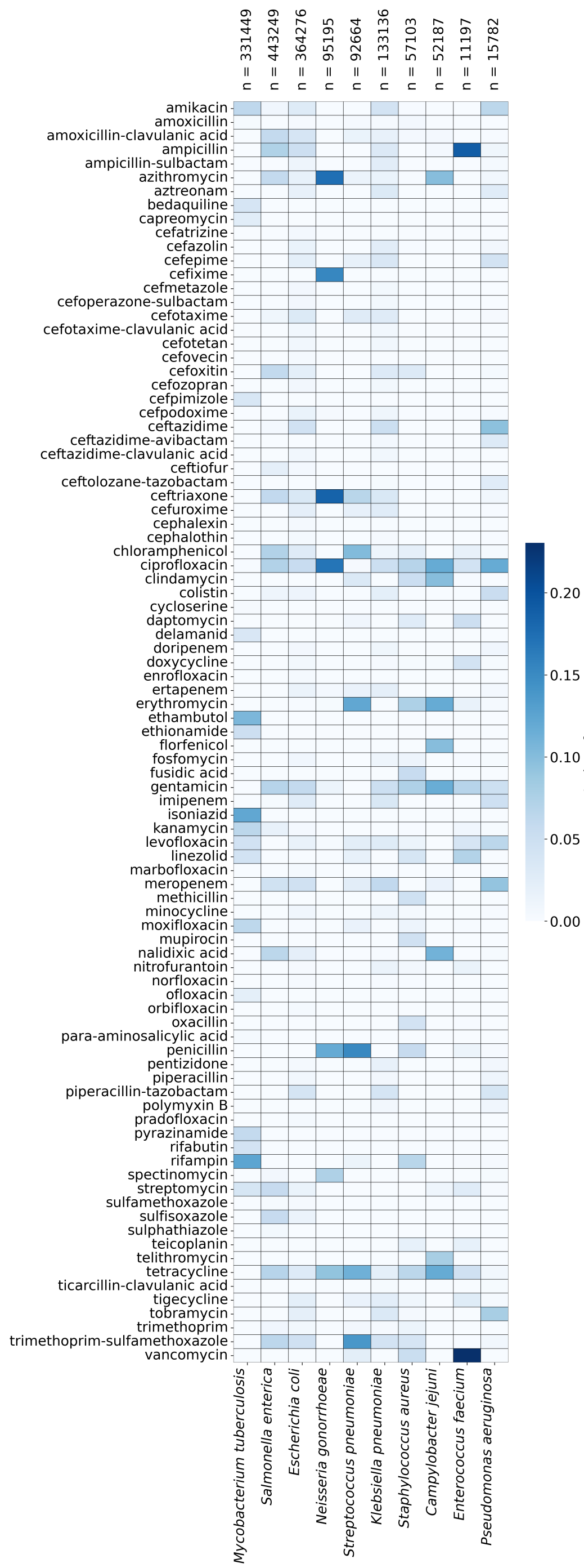

Figure S5: Heat map showing the antibiotic testing frequency (either measurement value, phenotype, or both) across the 10 most common bacterial species in CABBAGE. The colour of each cell represents the testing frequency for a specific pathogen-antibiotic pair, with dark blue indicating a high testing frequency and light blue indicating antibiotics that were rarely tested against the corresponding pathogen. The data was restricted to antibiotics represented by >=1,000 entries in CABBAGE.

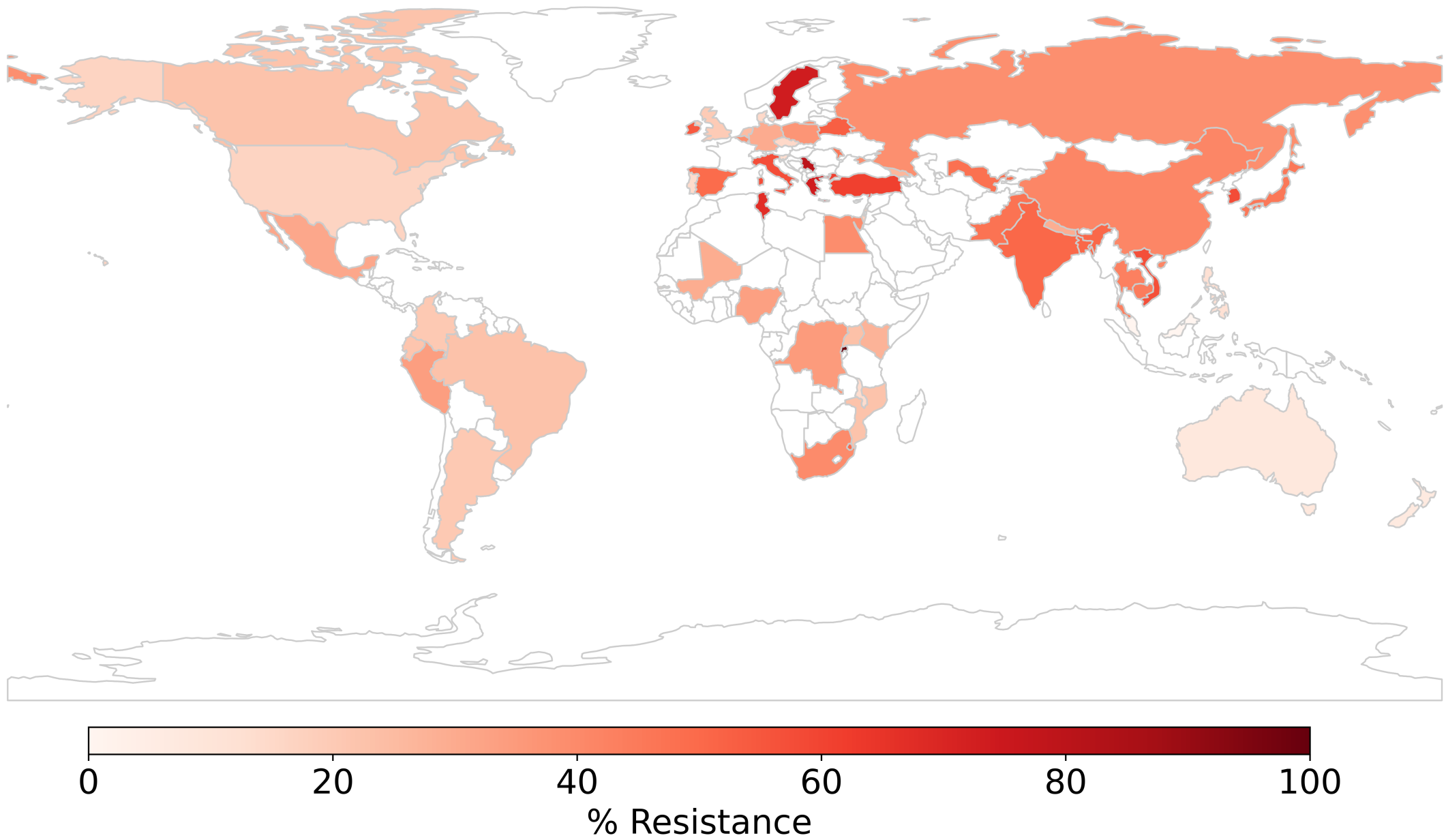

Figure S6: Antimicrobial resistance frequencies by country among phenotypically characterised isolates. Phenotypes derived from the original data source are shown, and countries with fewer than 500 entries are shown in white.

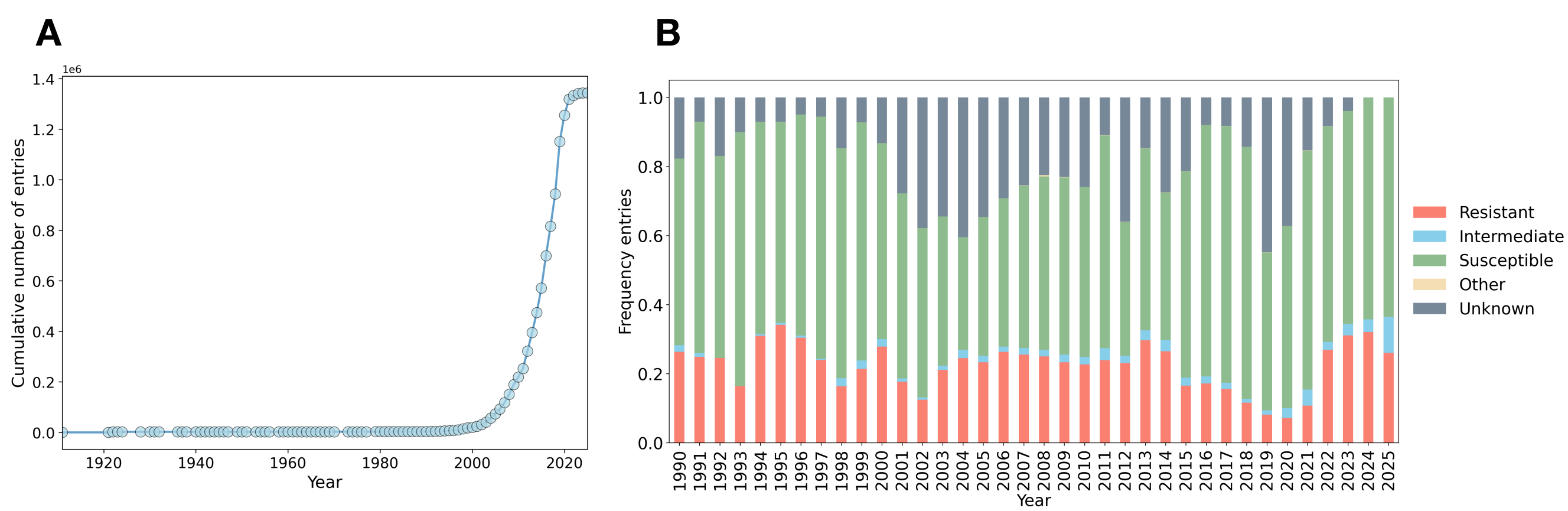

Figure S7: A) The cumulative number of entries (isolate-antibiotic pairs) over time (years), where the isolate collection date was available. B) The frequency of isolate-antibiotic pairs associated with each phenotype between 1990-2025. The category ‘other’ includes susceptible-dose dependent and non-susceptible.

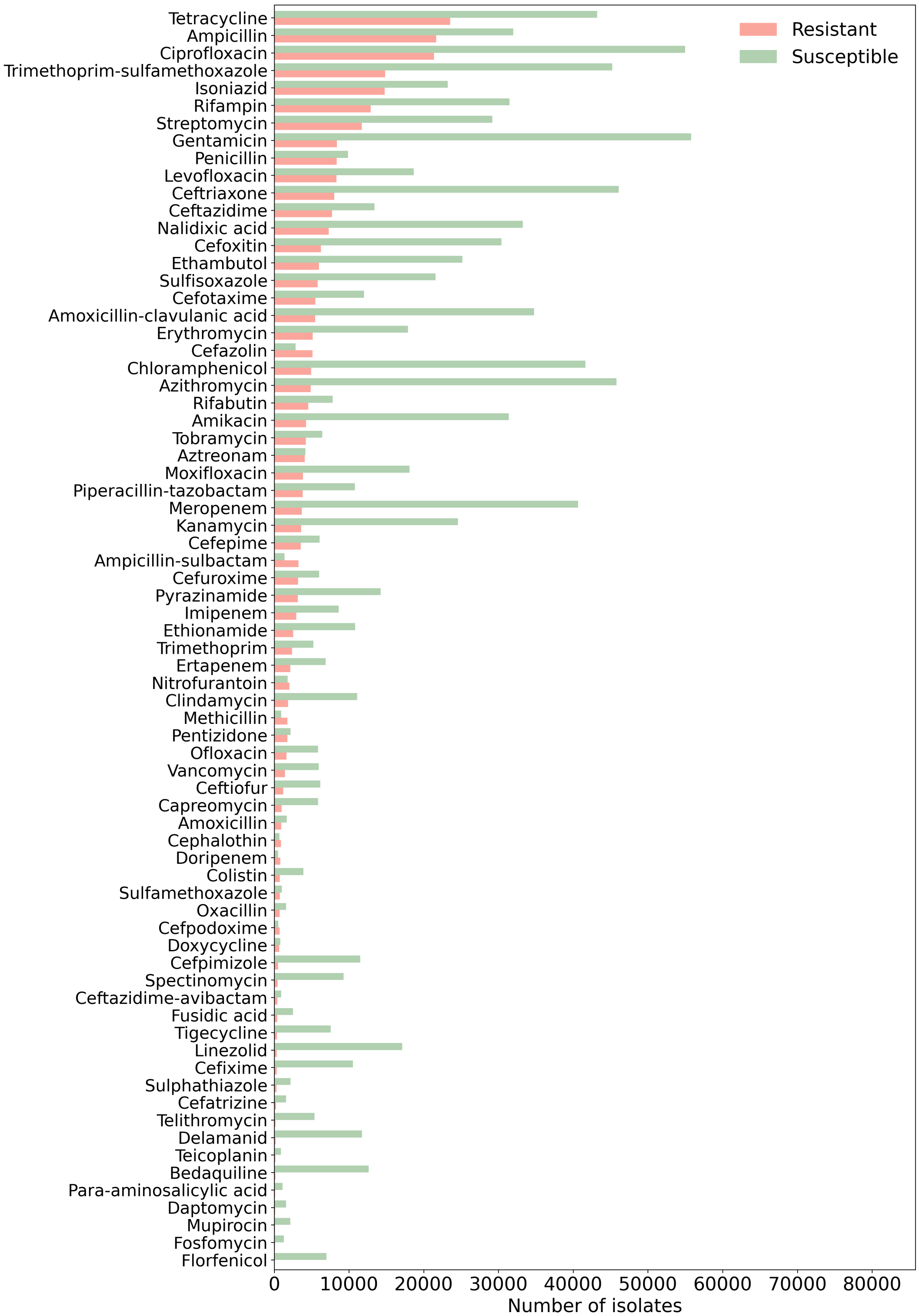

Figure S8: The number of resistant (red bars) versus susceptible (green bars) phenotypes is shown for each antibiotic. The data was restricted to those antibiotics with $\geq$ 1,000 isolates in CABBAGE.

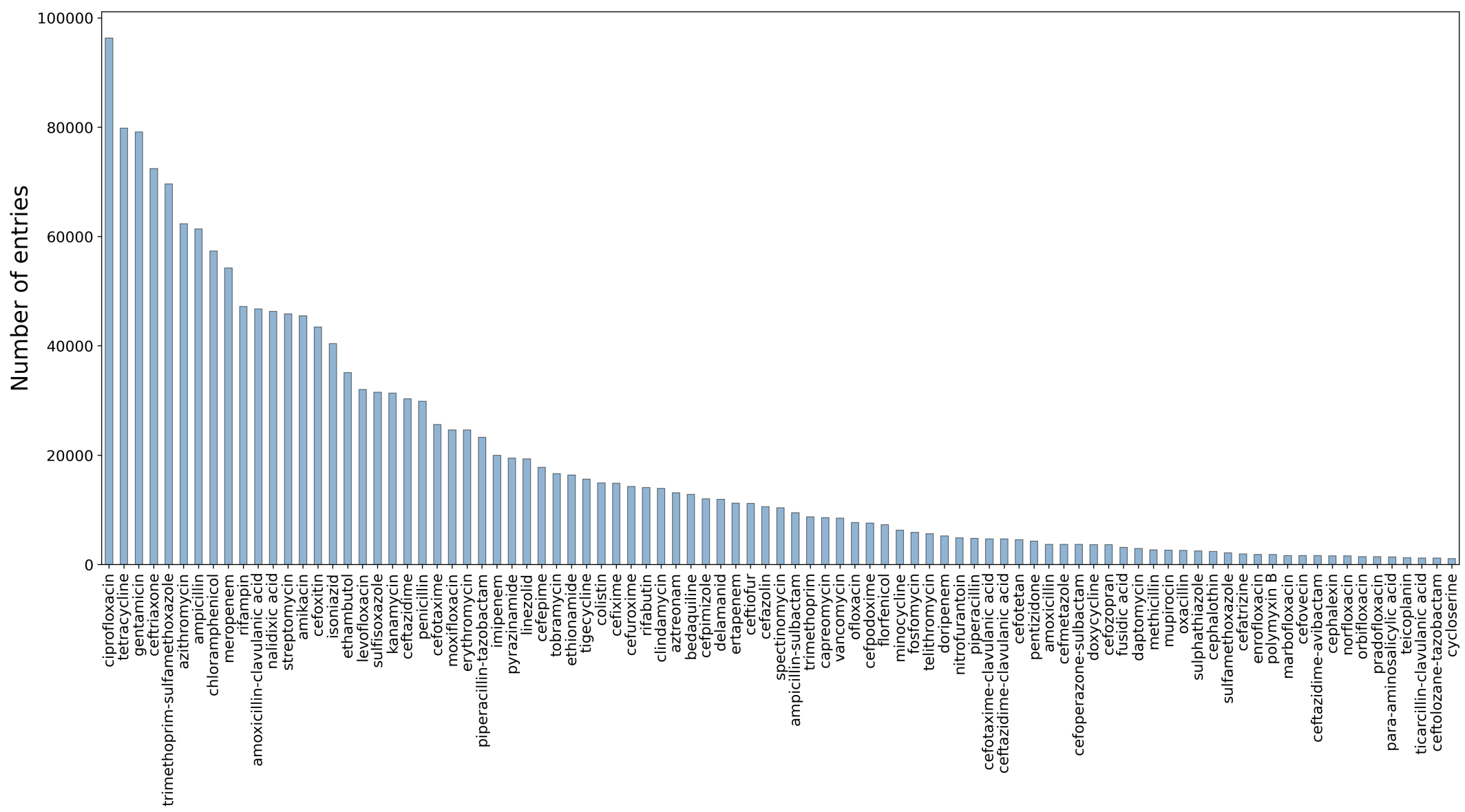

Figure S9: The number of isolates tested against each antibiotic. The data was restricted to antibiotics represented by $\geq$ 1,000 isolates in CABBAGE.

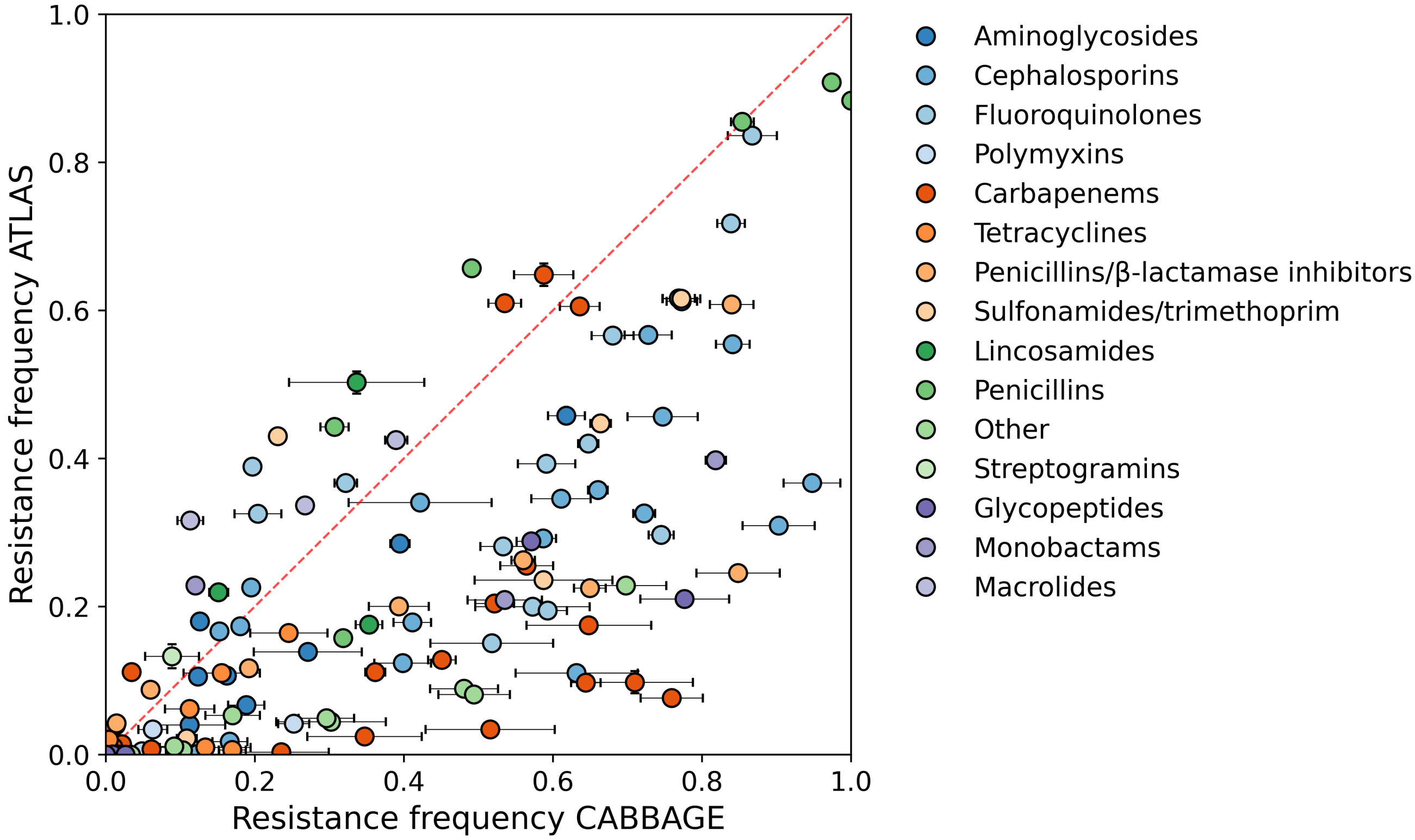

Figure S10: A scatter plot showing the frequency of resistance in CABBAGE and ATLAS, with each data point representative of a specific pathogen-antibiotic pair that is present in both datasets. The data has not been filtered by year, and only pathogen-antibiotic pairs with at least 100 entries in both CABBAGE and ATLAS are shown. Error bars represent 95% CI and colours are representative of different antibiotic classes. Note that the CABBAGE phenotypes presented here represent the phenotypes derived from the original datasets, rather than updated phenotypes.

REFERENCES

[1] Bramer W, de Jonge G, Rethlefsen M, et al. A systematic approach to searching: an efficient and complete method to develop literature searches. J Med Libr Assoc. 2018;106. http://dx.doi.org/10.5195/jmla.2018.283.

[2] Centers for Disease Control and Prevention (CDC). Centers for Disease Control and Prevention (U.S.), Antibiotic resistance threats in the United States, 2019 ; 2019. http://dx.doi.org/10.15620/cdc:82532.

[3] World Health Organisation, Country Office for India, Indian priority pathogen list; 2021. https://dbtindia.gov.in/sites/default/files/IPPL_final.pdf.

[4] VanOeffelen M, Nguyen M, Aytan-Aktug D, et al. A genomic data resource for predicting antimicrobial resistance from laboratory-derived antimicrobial susceptibility phenotypes. Briefings Bioinf. 2021;22:bbab313. https://doi.org/10.1093/bib/bbab313.

[5] Matamoros S, Hendriksen RS, Pataki B, et al. Accelerating surveillance and research of antimicrobial resistance an online repository for sharing of antimicrobial susceptibility data associated with whole-genome sequences. Microb Genomics. 2020;6:e000342. https://doi.org/10.1099/mgen.0.000342.

[6] Argimón S, Abudahab K, Goater RJE, et al. Microreact: visualizing and sharing data for genomic epidemiology and phylogeography. Microb Genomics. 2016;2:e000093. https://doi.org/10.1099/mgen.0.000093.

[7] David S, Reuter S, Harris SR, et al. Epidemic of carbapenem-resistant *Klebsiella pneumoniae* in Europe is driven by nosocomial spread. Nat Microbiol. 2019;4:1919-29. https://doi.org/10.1038/s41564-019-0492-8.

[8] Croucher NJ, Hanage WP, Harris SR, et al. Variable recombination dynamics during the emergence, transmission and 'disarming'of a multidrug-resistant pneumococcal clone. BMC Biol. 2014;12:49. https://doi.org/10.1186/1741-7007-12-49.

[9] Mutreja A, Kim DW, Thomson NR, et al. Evidence for several waves of global transmission in the seventh cholera pandemic. Nature. 2011;477:462-5. https://doi.org/10.1038/nature10392.

[10] Harris SR, Feil EJ, Holden MTG, et al. Evolution of MRSA During Hospital Transmission and Intercontinental Spread. Science. 2010;327:469-74. https://doi.org/10.1126/science.1182395.

[11] Wong VK, Baker S, Pickard DJ, et al. Phylogeographical analysis of the dominant multidrug-resistant H58 clade of *Salmonella Typhi* identifies inter- and intracontinental transmission events. Nat Genet. 2015;47:632-9. https://doi.org/10.1038/ng.3281.

[12] Do Nascimento V, Day MR, Doumith M, et al. Comparison of phenotypic and WGS-derived antimicrobial resistance profiles of enteroaggregative *Escherichia coli* isolated from cases of diarrhoeal disease in England, 201516. J Antimicrob Chemother. 2017;72:3288-97. https://doi.org/10.1093/jac/dkx301.

[13] Gentle A, Day MR, Hopkins KL, et al. Antimicrobial resistance in Shiga toxin-producing *Escherichia coli* other than serotype O157:H7 in England, 20142016. J Med Microbiol. 2020;69:379-86. https://doi.org/10.1099/jmm.0.001146.

[14] Berends MS, Luz CF, Friedrich AW, et al. AMR: An R Package for Working with Antimicrobial Resistance Data. Journal of Statistical Software. 2022;104:131. https://doi.org/10.18637/jss.v104.i03.

[15] Eldholm V, Monteserin J, Rieux A, et al. Four decades of transmission of a multidrug-resistant *Mycobacterium tuberculosis* outbreak strain. Nat Commun. 2015;6:7119. https://doi.org/10.1038/ncomms8119.

[16] McArthur AG, Waglechner N, Nizam F, et al. The Comprehensive Antibiotic Resistance Database. Antimicrob Agents Chemother. 2013;57:33483357. https://doi.org/10.1128/aac.00419-13.

[17] McLaughlin J, Lagrimas J, Iqbal H, et al. OLS4: a new Ontology Lookup Service for a growing interdisciplinary knowledge ecosystem. Bioinformatics. 2025;41:btaf279. https://doi.org/10.1093/bioinformatics/btaf279.
